# Most Ventral Pallidal Cholinergic Neurons Are Bursting Basal Forebrain Cholinergic Neurons with Mesocorticolimbic Connectivity

**DOI:** 10.1523/JNEUROSCI.0415-25.2026

**Published:** 2026-01-28

**Authors:** Dániel Schlingloff, Írisz Szabó, Éva Gulyás, Bálint Király, Réka Kispál, Marcus Stephenson-Jones, Balázs Hangya

**Affiliations:** ^1^MTA–HUN-REN KOKI Lendület “Momentum” Laboratory of Systems Neuroscience, HUN-REN Institute of Experimental Medicine, Budapest H-1083, Hungary; ^2^Department of Neuroscience, University of Copenhagen, Copenhagen DK-2200, Denmark; ^3^Institute of Artificial Intelligence, Medical University of Vienna, Vienna 1090, Austria; ^4^Sainsbury Wellcome Centre, University College London, London W1T 4JG, United Kingdom; ^5^Division of Neurophysiology, Center for Brain Research, Medical University of Vienna, Vienna 1090, Austria

**Keywords:** basal forebrain, cholinergic, diagonal band of Broca, learning, ventral pallidum

## Abstract

The ventral pallidum (VP) lies at the intersection of basal ganglia and basal forebrain circuitry, possessing attributes of both major subcortical systems. Basal forebrain cholinergic neurons (BFCNs) are rapidly recruited by reinforcement feedback and project to cortical and subcortical forebrain targets; in contrast, striatal cholinergic cells are local interneurons exhibiting classical “pause-burst” responses to rewards. However, VP cholinergic neurons (VPCNs) are less characterized, and it is unclear whether basal forebrain and striatal-type cholinergic neurons mix in the VP. Therefore, we performed anterograde and monotranssynaptic retrograde labeling, in vitro acute slice recordings and bulk calcium recordings of VPCNs in mice of either sex. We found that VPCNs broadly interact with the mesocorticolimbic circuit that processes rewards and punishments, targeting the basolateral amygdala, the medial prefrontal cortex, and the lateral habenula while receiving inputs from the nucleus accumbens, hypothalamus, central amygdala, bed nucleus of stria terminalis, and ventral tegmental area. Bulk calcium recordings revealed that VPCNs responded to rewards, punishments, and reward-predicting cues. Acute slice recordings showed that most VPCNs resembled the bursting type of BFCNs, while a few of them were of the regular rhythmic type, which differentiated most VPCNs from striatal cholinergic interneurons. These results were confirmed by in vivo electrophysiological recordings of putative VPCNs. We conclude that VPCNs show burst firing and specialized connectivity to relay aversive and appetitive stimuli to the reinforcement circuitry, possibly implicated in mood disorders and addiction.

## Significance Statement

The ventral pallidum (VP) is a special brain area, being part of both the basal ganglia system implicated in goal-directed behavior and the basal forebrain system implicated in learning and attention. It houses, among others, neurons that release the neurotransmitter acetylcholine. While these cholinergic neurons have distinct characteristics in other regions of the basal ganglia and basal forebrain, it is unclear whether those in the VP resemble one or the other or both. Here we demonstrate that they are closer to basal forebrain cholinergic neurons both anatomically and functionally, especially resembling a burst-firing subtype thereof. In accordance, we found that they convey information about aversive and appetitive stimuli to the reinforcement circuitry, possibly implicated in mood disorders and addiction.

## Introduction

The ventral pallidum (VP) is considered as the major output structure of the ventral basal ganglia (Maurice et al., 1997; Kupchik et al., 2015; Ahrens et al., 2016; Richard et al., 2016; Stephenson-Jones et al., 2020), thought to mediate the reinforcing and incentive properties of reward-predicting cues and rewards (Tindell et al., 2005; Tachibana and Hikosaka, 2012; Ahrens et al., 2018; Fujimoto et al., 2019; Ottenheimer et al., 2020a; Hegedüs et al., 2021) and drive reward-seeking behaviors (Smith et al., 2009; Richard et al., 2016, 2018; Stephenson-Jones et al., 2020; Ottenheimer et al., 2020b; Hernandez-Jaramillo et al., 2024). A study further suggested that the VP is monitoring information for upcoming choice behaviors, which it then relays to downstream decision-making areas (Ito and Doya, 2009).

At the same time, the VP is also categorized as part of the basal forebrain circuitry (Zaborszky et al., 2012; Faget et al., 2018), integrating limbic and cognitive signals. Indeed, Avila and Lin found that putative GABAergic VP neurons with a bursting phenotype resembled those of other basal forebrain regions and shared their salience-coding properties (Lin and Nicolelis, 2008), suggesting that salience information in the VP might be conveyed by afferents characteristic to the basal forebrain (Avila and Lin, 2014). In line with this, while reward-related signals in the VP were typically attributed to nucleus accumbens inputs (Kupchik et al., 2015; Root et al., 2015; Creed et al., 2016; Pardo-Garcia et al., 2019), a study found earlier and stronger reward value signals in the VP when performing a direct comparison with the accumbens (Ottenheimer et al., 2018), raising the possibility that other afferents may play a major role in these rapid reward responses (Ottenheimer et al., 2018; Soares-Cunha and Heinsbroek, 2023). Another study found that somatostatin-expressing GABAergic VP neurons participate in controlling cortical gamma oscillations (Espinosa et al., 2019), which is a well-established function of the basal forebrain's cortical projections, implicated in controlling attention and arousal (Kim et al., 2015; Yang et al., 2017; Király et al., 2023).

Interpreting the VP as a basal ganglia output has initially directed the focus to VP GABAergic neurons (van den Bos and Cools, 1991; Soares-Cunha and Heinsbroek, 2023); however, the VP contains considerable glutamatergic and cholinergic populations that have been addressed more recently (Faget et al., 2018; Stephenson-Jones et al., 2020; Farrell et al., 2021). Stephenson-Jones and colleagues found that both GABAergic and glutamatergic VP neurons can drive movement, but they are active in opposite valance contexts: GABAergic cells represent positive values and drive approach, while glutamatergic neurons represent negative values and drive avoidance (Stephenson-Jones et al., 2020). Similar results were found in the context of cocaine seeking (Heinsbroek et al., 2020), and it was shown that distinct inhibitory and excitatory VP projections mediate different aspects of depression-like symptoms (Knowland et al., 2017) and alcohol relapse (Prasad et al., 2020). Differential nucleus accumbens inputs to VP GABAergic versus glutamatergic neurons were proposed to at least partially underlie the above differences (Neuhofer and Kalivas, 2023).

Comparably less is known about VP cholinergic neurons (VPCNs; Walaas and Fonnum, 1979; Zaborszky et al., 2012; Root et al., 2015). Cholinergic-specific VP lesions increased active coping mechanisms in fearful situations in mice (Akmese et al., 2023) and optogenetic stimulation of VP to basolateral amygdala (BLA) cholinergic projections reduced pain thresholds and increased depression-like behaviors (Ji et al., 2023). Kim and colleagues found that this projection mostly coded aversive information, while a distinct set of cholinergic neurons represented appetitive cues in the context of odor discrimination (Kim et al., 2024).

However, a comprehensive account of the basal forebrain cholinergic population, including input–output mapping and their functional positioning along the basal ganglia—basal forebrain axis is missing, limiting the understanding of VP circuitry and functions. We fill this knowledge gap by revealing VPCN input–output connectivity including long-range cortical projections and showing that VPCNs show bursting responses to task-relevant salient stimuli.

## Materials and Methods

### Animals

For targeted in vitro electrophysiological characterization of VPCNs and striatal cholinergic interneurons (CINs), fluorophor expression in cholinergic neurons was driven by crossing ChAT-Cre and Ai32 [*n* = 4; 2/4 males; Postnatal Day (P)40–60] or ChAT-Flp and Ai213 mice (*n* = 1 male; P40). ChAT-Cre mice were used for the characterization of regular rhythmic and bursting basal forebrain cholinergic neurons (Reg-BFCNs and Burst-BFCNs; *n* = 12; 7/12 males; P50–150). ChAT-Cre mice were used for anterograde (*n* = 6; 4/6 males; P50–100) and retrograde (*n* = 6; 3/6 males; P50–100) anatomical tracings. For the fiber photometry measurements, we used ChAT-Cre mice (*n* = 22; 14/22 males; P90–120). All experiments were conducted according to the regulations of the European Community's Council Directive of November 24, 1986 (86/609/EEC); experimental procedures were reviewed and approved by the Animal Welfare Committee of the Institute of Experimental Medicine, Budapest, and the Committee for Scientific Ethics of Animal Research of the National Food Chain Safety Office.

### Surgeries and viruses

Mice were anesthetized using a ketamine–xylazine solution (83 mg/kg ketamine and 17 mg/kg xylazine, prepared in 0.9% saline). After shaving and disinfecting the scalp with Betadine, the skin and subcutaneous tissues were numbed topically with lidocaine spray. The mice were then positioned in a stereotaxic frame (David Kopf Instruments), and their eyes were protected with Corneregel eye ointment (Bausch & Lomb). A sagittal incision was made in the skin using a surgical scalpel, exposing the skull, which was then cleaned. A craniotomy was drilled above the targeted area. For anterograde and retrograde tracings the craniotomy was opened above the VP (anteroposterior 0.5 mm; lateral 1 mm). Virus injections were performed for anterograde and retrograde tracing using a stereotaxic frame and a programmable nanoliter injector (Drummond Nanoject III). For anterograde tracing, AAV2/5.EF1a.Dio.hChR2(H134R)-eYFP.WPRE.hGH (Addgene; titer ≥1 × 10^13^ vg/ml) was injected into the VP at a dorsoventral depth of 4.20 mm (20–30 nl). Retrograde tracing involved sequential injections of AAV2/9-Syn-FLEX-nGToG-WPRE3 (50 nl, catalog #BA-96, VCF of the Charité) and, after a 4 week interval, pSADB19dG-mCherry (100 nl, catalog #BR-001, VCF of the Charité) at the same dorsoventral depth. Anterograde virus injections were allowed a 4 week expression period, whereas retrograde tracings included a 9 d expression period following the rabies injection. For targeted in vitro electrophysiological characterization of Reg- and Burst-BFCNs, AAV2/5-EF1a-DIO-hChR2(H134R)-mCherry-WPRE-HGHpA was injected either into the caudal NB (anteroposterior −0.9 mm, lateral 2.2 mm, 3–4 dorsoventral levels between 3.3 and 5 mm) or the horizontal limb of the diagonal band of Broca (HDB; see also Table S1 for all abbreviations; anteroposterior 0.75 mm, lateral 0.6 mm, 2 dorsoventral levels between 4.5 and 5.5 mm; Laszlovszky et al., 2020). For fiber photometry experiments, mice were injected with AAVD7/2-CAG-hsyn-jGCaMP8m(rev)-dlox-WPRE-SV40r(A) (HDB and VP, 150 nl each side; HDB, anteroposterior 0.75 mm, lateral −0.60 mm; dorsoventral −4.7 mm; VP, anteroposterior −0.61 mm, lateral 1.00 mm; dorsoventral −4.5 mm). During fiber photometry surgeries, injections were followed by the bilateral implantation of 400 μm core diameter optic fibers with ceramic ferrules (HDB, anteroposterior 0.75 mm, lateral −2.10 mm, dorsoventral −4.5 mm; 20° lateral angle; VP, anteroposterior −0.61 mm, lateral 1.00 mm, dorsoventral −4.3 mm; 0° lateral angle). The implant was secured to the skull with Super-Bond (Sun Medical) and dental cement. Mice received analgesics (buprenorphine, 0.1 mg/kg) and local antibiotics (gentamycin) and were allowed 10 d of recovery before starting behavioral training. All experiments were concluded by transcardial perfusion, and mouse brains were processed for further immunohistology experiments (see below).

### Behavioral training for fiber photometry

Mice were trained on a head-fixed auditory pavlovian conditioning task. The behavioral setup was custom-built to allow millisecond precision control of stimulus and reinforcement timing (Solari et al., 2018). Mice were subjected to a standard water restriction protocol prior to training and earned small water rewards (4 μl) during conditioning. Two pure tones of different pitch (4 and 12 kHz, balanced across *n* = 10 and *n* = 12 mice; duration, 1 s) predicted water reward or air-puff punishment with 90% probability (10% omissions). Training started with Stage 0, in which mice listened to Cue 1 that was paired with 90% water rewards and 10% omissions. In Stage 1, we introduced Cue 2 (25% of all trials) but without the air puffs. In Stage 2, Cue 2 was paired with 90% air-puff punishments and 10% omissions. In Stage 3, the proportion of Cue 2 trials was raised to 40%. In the final stage (Stage 4), the two cue tones were presented in a randomized 50–50% ratio. All tones were set to 65 dB SPL. After the onset of the tone, mice could lick a waterspout, and individual licks were recorded by detecting when their tongues interrupted an infrared beam. Following a 400–600 ms poststimulus delay, the scheduled outcome (water, air-puff, or omission) was delivered in a pseudorandomized order based on the cue contingencies. Each new trial began after the animal refrained from licking for a minimum of 2.5 s. A foreperiod of 2.5–5.5 s, drawn from a truncated exponential distribution, preceded each stimulus to prevent temporal expectations. Trials were restarted if the mouse licked during this foreperiod. Task control was handled by the Bpod behavioral system (Sanworks). Air puffs, 200 ms in duration, were delivered at 15 psi pressure, which stimulus was reported as aversive for head-fixed mice (Najafi et al., 2014; Hangya et al., 2015).

### Fiber photometry imaging

Dual-channel fiber photometry was used to monitor bilateral calcium activity, with fluorescence signals visualized throughout training sessions using the Doric Studio Software (Doric Neuroscience). Two LED sources (465 and 405 nm) were used in combination with fluorescent Mini Cubes (iFMC4, Doric Neuroscience). Amplitude modulation of the LEDs was achieved via a two-channel driver (LEDD_2, Doric Neuroscience), with 465 nm light modulated at 208 Hz and 405 nm light modulated at 572 Hz. The light was delivered to 400 µm patch cord fibers and connected to optical implants during the sessions. The same fibers were used to collect the fluorescence emitted from the tissue, which was detected by 500–550 nm photodetectors integrated into the Mini Cubes. Signals were sampled at 12 kHz, digitally decoded, and saved in *.csv format for later analysis.

### Perfusion

Mice were anesthetized with 2% isoflurane followed by an intraperitoneal injection of a mixture of ketamine–xylazine and promethazinium–chloride (83, 17 and 8 mg/kg, respectively). After achieving deep anesthesia, mice were perfused transcardially (by placing the cannula into the ascending part of the aorta via an incision placed on the left ventricle wall) with saline for 2 min, followed by 4% paraformaldehyde (PFA) solution for 40 min and then saline for 10 min. After perfusion, mice were decapitated, and brains were carefully removed from the skull and postfixed in PFA overnight.

### Track verification for fiber photometry

A block containing the full extent of the HDB and VP was prepared, and 50-µm-thick sections were cut using a Leica 2100S vibratome. All attempts were made to section parallel to the canonical coronal plane to aid track reconstruction efforts. All sections that contained the tracks were mounted on slides in Aquamount mounting medium. Epifluorescense images of the sections were taken with a Nikon C2 confocal microscope or Pannoramic Midi Slidescanner. Atlas images were aligned to fluorescent images of the brain sections showing the fiber tracks and green fluorescent labeling in the target area. Only those recordings that were unequivocally localized to the HDB and VP were analyzed in this study.

### Anterograde and retrograde tracing

In case of anterograde tracing experiments, coronal sections of 50 µm thickness were cut by a vibratome (Leica VT1200S). Sections were extensively washed in 0.1 M PB and TBS and blocked in 1% human serum albumin (HSA; Sigma-Aldrich) solution for 1 h. Then, sections were incubated in primary antibodies against eGFP (Thermo Fisher Scientific, catalog #A10262, 1:2,000, raised in chicken; Table S2) for 48–60 h. Sections were rinsed three times for 10 min in TBS; secondary fluorescent antibodies were applied overnight (anti-chicken Alexa Fluor 488, Jackson Immunoresearch Laboratories, catalog #703-545-155, 1:1,000; Table S2). Sections were rinsed in TBS and 0.1 M PB and mounted on slides in Aquamount mounting medium (BDH Chemicals). Sections containing the VP were incubated in primary antibody against ChAT (Synaptic Systems, catalog #297013, 1:500, raised in rabbit; Table S2) and anti-rabbit Alexa Fluor 594 secondary antibody (Thermo Fisher Scientific, catalog #A21207, 1:500; Table S2). After identifying brain regions with strong axonal density, fluorescent images were taken with a Nikon A1R Confocal Laser Scanning Microscope. In the target areas of the VPCNs, three fluorescent *z*-stack images were captured at 20× magnification from each animal in each region using a standardized volume. These stacks were projected into single planes, and axonal density was quantified using the open-source software Ilastik, which is specifically designed for machine learning–based image processing (Berg et al., 2019). In our analysis, we used the Pixel Classification workflow, where axons were manually annotated to train a classifier. This classifier then generated probability maps, assigning each pixel a likelihood of representing an axon. These probability values were subsequently used to estimate axonal densities for each sampled region. Compared with commonly used approaches that rely on mean pixel brightness to estimate axonal density, this method provides a more reliable and biologically meaningful measure, as it distinguishes axonal structures from background signal and imaging noise.

In case of retrograde tracing experiments, coronal sections of 50 µm thickness were cut by a vibratome (Leica VT1200S). Sections were extensively washed in 0.1 M PB and TBS and blocked in 1% HSA (Sigma-Aldrich) solution for 1 h. Then, sections were incubated in primary antibodies against eGFP (Thermo Fisher Scientific, catalog #A10262, 1:2,000, raised in chicken; Table S2) and mCherry (Biovision, catalog #5993-100, 1:1,000, raised in rabbit; Table S2) for 48–60 h. Sections were rinsed three times for 10 min in TBS; secondary fluorescent antibodies were applied overnight (anti-chicken Alexa Fluor 488, Jackson ImmunoResearch Laboratories, catalog #703-545-155, 1:1,000, anti-rabbit Alexa Fluor 594, Thermo Fisher Scientific, catalog #A21207, 1:500; Table S2). Sections were rinsed in TBS and 0.1 M PB and mounted on slides in Aquamount mounting medium (BDH Chemicals). Every second section was sampled to measure and estimate the number of transsynaptically labeled input cells using a Zeiss Axioplan2 epifluorescent microscope and a Pannoramic Digital Slide Scanner (3DHISTECH Kft.). We quantified labeled cells across all brain regions containing input neurons. To control for variability in viral spread, we normalized the data by calculating the percentage of labeled neurons in each input region relative to the total number of labeled neurons for that animal. These percentages were then averaged across animals. This approach reduces potential confounds related to the injection site size or viral efficiency, as the analysis relies on relative rather than absolute labeling.

### Acute in vitro slice preparation

Mice were decapitated under deep isoflurane anesthesia, and the brains were rapidly removed and placed in ice-cold cutting solution, precarbogenated (95% O_2_–5% CO_2_) for at least 30 min before use. The cutting solution consisted of the following (in mM): 205 sucrose, 2.5 KCl, 26 NaHCO_3_, 0.5 CaCl_2_, 5 MgCl_2_, 1.25 NaH_2_PO_4_, and 10 glucose. Coronal slices, 300 μm thick, were prepared using a vibratome (Leica VT1200S). Following acute slice preparation, slices were transferred to an interface-type holding chamber for at least 1 h of recovery. This chamber contained artificial cerebrospinal fluid (ACSF) solution maintained at 35°C, which gradually cooled to room temperature. The ACSF solution consisted of the following (in mM): 126 NaCl, 2.5 KCl, 26 NaHCO_3_, 2 CaCl_2_, 2 MgCl_2_, 1.25 NaH_2_PO_4_, and 10 glucose, saturated with carbogen gas as described above.

### In vitro electrophysiology recordings

Recordings were performed under visual guidance using a Nikon Eclipse FN1 microscope with infrared differential interference contrast optics. The flow rate of the ACSF was 4–5 ml/min at 30–32°C (Supertech Instruments). Patch pipettes were pulled from borosilicate capillaries [with inner filament, thin-walled, outer diameter (OD) 1.5] with a PC-10 puller (Narishige). Pipette resistances were 3–6 MΩ when filled with intrapipette solution. The composition of the intracellular pipette solution was as follows (in mM): 110 d-gluconic acid potassium salt, 4 KCl, 20 HEPES, 0.1 EGTA, 10 phosphocreatine di(Tris) salt, 2 ATP magnesium salt, and 0.3 GTP sodium salt, with 0.2% biocytin, adjusted to pH 7.3 using KOH and with osmolarity of ∼295 mOsm/L. Recordings were performed with a Multiclamp 700B amplifier (Molecular Devices), digitized at 10 or 20 kHz with Digidata analog–digital interface (Molecular Devices) and recorded with pClamp11 Software suite (Molecular Devices). Cholinergic neurons expressing GFP or mOrange were visualized with the aid of LED light sources (Prizmatix) integrated into the optical light path of the microscope and detected with a CCD camera (Andor Zyla, Oxford Instruments). We applied a somatic current injection protocol containing a 3-s-long, incremental “prepolarization” step followed by a positive square pulse (1 s), to elicit spiking starting from different membrane potentials as in Laszlovszky et al. (2020). Furthermore, we applied a simple step protocol consisting of a series of hyperpolarizing and depolarizing steps, each lasting 1 s, to further determine the spiking characteristics of distinct cholinergic cell types.

### Immunohistochemical identification of in vitro recorded cholinergic cells

After acute slice electrophysiology experiments, brain sections were fixed overnight in 4% PFA. Sections were extensively washed in 0.1 M PB and TBS and blocked in 1% HSA (Sigma-Aldrich) solution for 1 h. Then, sections were incubated in primary antibody against ChAT (Synaptic Systems, catalog #297013, 1:500; Table S2) for 48–60 h. This step was followed by thorough rinse with TBS (3 × 10 min) and overnight incubation with a mixture of anti-rabbit Alexa Fluor 594 secondary antibody (Thermo Fisher Scientific, catalog #A21207, 1:500; Table S2) and streptavidin-A488 (Invitrogen, catalog #S11223, 1:1,000). We used 0.1% Triton X-100 detergent through every incubation step due to the thickness of the brain section. Finally, sections were washed in TBS and PB, mounted on microscopy slides, covered with Vectashield (Vector Laboratories) and imaged with a Nikon A1R confocal laser scanning microscope.

### Analysis of in vitro experiments

All in vitro data were processed and analyzed offline using Python 3. Spike delay was defined as the interval between the start of the 1 s positive current injection step and the peak time of the first action potential (AP) and was calculated using the “prepolarization” protocol. Burst frequency was determined from the subsequent three interspike intervals (ISIs). Autocorrelograms (ACGs) for each cell were computed using spikes evoked by simple step protocols and were smoothed with a 5 ms moving average. A comprehensive set of electrophysiological features was extracted using the Electrophys Feature Extraction Library (Ranjan et al., 2024), including afterhyperpolarization properties, AP waveform metrics (amplitude, width, duration, rise/fall dynamics, and inter-AP differences), ISI statistics, spike count and timing measures, as well as passive membrane properties (e.g., voltage deflection, sag, input resistance, and decay constants). These features were derived from the first current injection step that elicited at least four APs in simple step protocols. The resulting dataset was used for the low-dimensional projection with Uniform Manifold Approximation Project (UMAP; McInnes et al., 2018).

### Analysis of in vivo electrophysiology data

#### Recording, spike sorting, and optotagging

We used electrophysiology recordings collected in Stephenson-Jones et al. (2020). In vivo recordings were conducted using custom-built screw–driven microdrives with tetrodes attached to a 50 μm optic fiber (Kvitsiani et al., 2013). Broadband signals were filtered between 0.2 and 8,500 Hz and recorded at a 25 kHz sampling rate. Next, the acquired signals were bandpass-filtered between 300 and 5,000 Hz for spike detection, and spike waveforms were sorted offline using MClust v3.5 (A.D. Redish). Well-isolated units with isolation distance over 20 and L-ratio under 0.1 were included based on amplitude and waveform energy features (Schmitzer-Torbert et al., 2005). Putative GABAergic or glutamatergic neurons in the VP were identified using ArchT-based optotagging (Courtin et al., 2013) in GAD2-IRES-Cre and Vglut2-Cre mice, respectively. After behavioral recordings, green laser pulses (532 nm, 200 ms) were delivered every 5 s for 100 trials. Neurons were considered tagged if their firing was rapidly suppressed (<10 ms latency) and stayed below 0.5 Hz during stimulation. Hierarchical clustering was performed on the first three principal components of neuronal responses to rewards and punishments as in Cohen et al. (2012), which identified four distinct functional classes. All identified glutamatergic neurons belonged to Type 2 while all identified GABAergic neurons belonged to Types 3 and 4. Thus, Type 1 contained putative cholinergic neurons—the only class that was characterized by activation after both rewards and punishments. Although an unambiguous separation of cholinergic neurons based on simple electrophysiological signatures has not been reported, they form separate principal component clusters in conditioning tasks featuring rewards and punishments that allows a reliable separation (Hangya et al., 2015), similar to the midbrain dopaminergic cell type (Cohen et al., 2012).

#### Eyeblink tracking

Data from Stephenson-Jones et al. (2020) were used. Briefly, a CMOS camera (QSICC2) was used to track eyeblinks. Videos were analyzed offline using the EthoVision XT software (Noldus). Oval regions of interest (ROIs) surrounding the eye were drawn manually and pixels darker than the background (corresponding to the eye) were detected. A threshold number of such pixels were used to define a blink.

#### Autocorrelation analysis

Data were processed in MATLAB R2018a (MathWorks). ACGs were computed at 0.5 ms resolution and smoothed using a 2.5 ms (five-point) moving average for visualization. Individual ACGs were normalized to their mean values, sorted by Burst Index or refractory period, and averaged per group. The Burst Index was calculated as the difference between the maximum ACG at 0–25 ms and the mean ACG at 180–200 ms, normalized by the larger of these two values, yielding an index between −1 and 1 [modified from Laszlovszky et al. (2020) based on the slower bursts of VPCNs]. The Theta Index was calculated based on the difference between the mean ACG values within a ±25 ms window around the 5–10 Hz theta peak (100–200 ms lags). Refractory periods were estimated by identifying low-probability spiking intervals from the ACGs, using a 10 ms moving average to find the half-height point of the ACG's central trough. This provided a measure of relative refractory periods rather than absolute spike repolarization (Royer et al., 2012; Laszlovszky et al., 2020).

#### Analysis of event-related firing rate changes

First, we searched for minimal/maximal firing rates as minimum/maximum values of perievent time histograms (PETHs) within 500 ms from rewards/punishments. For comparison, baseline firing was determined as the mean firing rate from the 500 ms window prior to rewards/punishments. Next, the time course of inhibition/activation was assessed by crossings of the half-distance between the extreme and the baseline before and after the minimum/maximum. This temporal window of inhibition/activation was then used to find corresponding intervals around local extremes in the baseline period. Spike counts in these baseline periods and spike counts in the previously determined inhibition/activation windows were then compared using one-sided Mann–Whitney *U* test (due to the asymmetric null hypothesis in each analysis). Significant firing rate changes were evaluated at *p* < 0.01 to keep the false-positive rate low. If both activation and inhibition reached significance, the earlier one was designated as the primary response.

### Analysis of fiber photometry recordings

#### Preprocessing

MATLAB R2018a was used to process fiber photometry data [following the procedures described in Lerner et al. (2015) and Hegedüs et al. (2023)]. Animals with sufficient viral expression in the target region, as well as successful surgical targeting that resulted in measurable fluorescent signals were included in the analyses, resulting in *n* = 21 mice for VP-specific analyses, *n* = 16 mice for HDB-specific analyses, and *n* = 15 mice for VP–HDB comparisons. The fluorescence signals were digitally filtered below 20 Hz using a low-pass Butterworth filter to remove high-frequency noise. The delta fluorescence (d*F*/*F*) signal was computed by fitting a least-squares regression to the 405 nm isosbestic control signal and aligning its baseline with that of the 465 nm calcium-dependent signal (f465). The normalized 405 nm signal (f405, fitted) was then subtracted from the 465 nm signal as follows: d*F*/*F* = (f465 − f405, fitted) / f405, fitted × 100, to account for motion artifacts and autofluorescence. Slow baseline decay was corrected with a 0.2 Hz high-pass filter. The d*F*/*F* signals were *Z*-scored relative to the mean and standard deviation of a baseline period (2 s before cue onset) for each trial.

#### PETHs

The normalized photometry traces were averaged across trials. The analysis included only the last five sessions where Cue 1 and Cue 2 occurred with equal (0.5) probabilities. Response maxima, along with latency, duration, and area under the curve (AUC), were computed as follows. Two time windows were defined for response (1 s relative from trigger) and baseline (2 s before stimulus start), respectively. Analyses were run for rewarded and punished trials, allowing for within-animal comparison across conditions. For each session, the calcium trace was *z*-scored using a baseline window (2 s) measured during ITIs: *z* = [trace − mean(baseline)] / SD(baseline). Peak value: the largest peak within the 1 s analysis window was identified using MATLAB's max function. AUC: the area under the signal was computed around the main peak in a window between the half-maximum locations by summing the d*F*/*F* values and dividing by the sampling rate. Duration of the response: the temporal width (in ms) of the signal at half-maximum was used to define response duration. Latency: the latency (in ms) of the largest peak was determined relative to the trigger event. In an additional analysis, Cue 2 responses were further characterized in a trialwise manner by comparing the maximum response value (0–0.5 s relative to stimulus) to baseline fluctuations (−0.5 s to 0 relative to stimulus), and latency and duration were calculated only for animals showing a significant increase relative to baseline.

#### Cross-correlation analysis

Sessions containing both cue types were included in the analysis. Cross-correlations (CCR) between two photometry signals (VP and HDB) were computed at the maximal time resolution allowed by the sampling rate (12,048 Hz) using MATLAB's built-in xcorr.m function, normalized to the autocorrelations at lag 0 (i.e., the signal magnitudes) as follows:

$${\hat{R}}_{xy,\mathrm{norm}}(t)=\frac{1}{\sqrt{{\hat{R}}_{xx}(0){\hat{R}}_{yy}(0)}}{\hat{R}}_{xy}(t),$$

where ${\hat{R}}_{xy}(t)$ is the CCR of the time series *x* and *y* at lag *t*. The CCR were calculated over the full length of the signal for each session. To reject common mode noise, the central ±20 ms window around 0 ms lag was excluded from the analysis. The resulting CCR curves were then restricted to a ±10 s window and averaged across sessions. Maximal CCR values as well as the time lag of maximal correlation were calculated for each animal.

### Analysis of pupil dynamics

To monitor pupil dynamics during behavioral training, we used a Flea3 FL3-U3-32S2M camera focused on the mouse's eye. Video capture was synchronized with the fiber photometry recording through TTL signals, with a TTL pulse sent at the beginning of each frame and recorded at 59 FPS. The videos were analyzed offline using DeepLabCut (Mathis et al., 2018), which was trained to track pupil edges at three diagonal points and eyelid positions. Pupil diameter was calculated as the mean distance between the three diagonal points and interpolated to match the sampling rate of the fiber photometry data. Calcium transient peaks recorded in either the VP or the HDB were used to calculate VP/HDB activity-evoked changes in the pupil size. Transfer entropy (TE) values were computed on the *z*-scored, downsampled, and discretized VP/HDB and pupil time series using the PyInform Python library for information-theoretic measures of time series data. During discretization, the continuous data were divided into 200 equally spaced bins, and each data point was assigned to its corresponding bin.

### Statistical analysis

We estimated the sample size before conducting the study based on previous publications (Laszlovszky et al., 2020; Hegedüs et al., 2023) and corresponding statistical power estimations (https://github.com/hangyabalazs/statistical-power). The study did not involve separate experimental groups, so randomization and blinding were not relevant to the study. Automated data analysis was conducted independently of neuron identity. For neurons with >50,000 spikes, ACG calculation was capped at 50,000 spikes to avoid memory limitations. Comparisons between conditions were performed using nonparametric tests to avoid assumptions on normality, which could not be confirmed statistically. The Wilcoxon signed-rank test was used for paired samples, while the Mann–Whitney *U* test was used for unpaired comparisons. PETHs were presented as mean ± SE, while box plots showed median, interquartile range, and nonoutlier range, with all data points displayed.

## Results

### Input–output connectivity of VPCNs reveal broad connections with the mesocorticolimbic circuit

Mapping of afferent and efferent connectivity of BFCNs have been carried out for the broadly defined basal forebrain (Do et al., 2016; Hu et al., 2016); however, these experiments did not include specific VP injections. Additionally, cholinergic output connectivity was determined for the substantia innominata (SI), HDB, and medial septum (MS) regions (Saper, 1984; Zaborszky et al., 2012; Agostinelli et al., 2019) but not for the VP.

**Figure 1.**
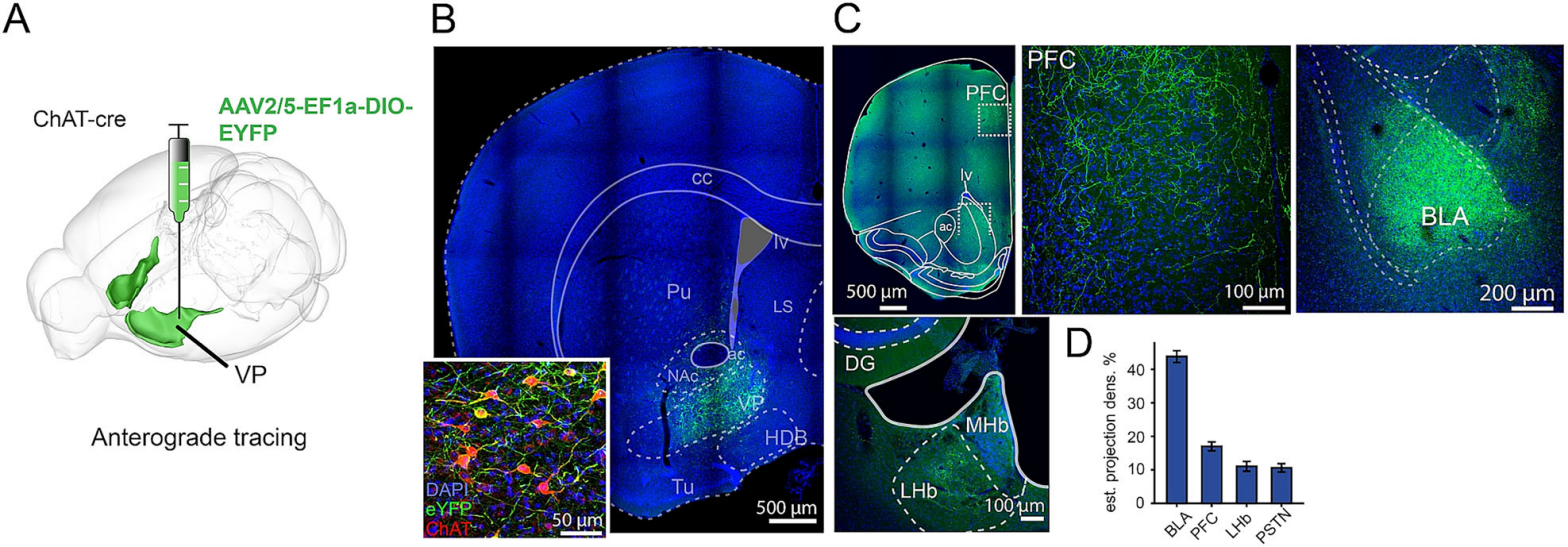
VPCNs innervate the mesocorticolimbic circuit. ***A***, Schematic illustration of an anterograde tracer virus injection into the VP of a ChAT-Cre mouse. See reconstructed injection sites in Figure S1*A*. ***B***, Fluorescent image of the injection site, showing eYFP (green) and DAPI (blue) labeling. ***C***, Fluorescent images showing the main target areas innervated by VPCNs, including the prefrontal cortex (PFC), the basolateral amygdala (BLA), and the lateral habenula (LHb); green, eYFP; blue, DAPI. ***D***, Estimated projection density in the primary output regions of VPCNs, expressed as a percentage of labeled axons (*n* = 6 mice). Bars and error bars indicate mean ± SEM (BLA, 43.81 ± 1.77%; PFC, 17.00 ± 1.33%; LHb, 11.04 ± 1.47%; PSTN, 10.59 ± 1.26%).

To fill this gap, we first performed anterograde tracing of VPCNs by injecting AAV2/5-EF1a-DIO-EYFP in the VP region of ChAT-Cre mice (Fig. 1*A*). We screened for the major anterograde projections of VPCNs and then acquired high-resolution confocal images to assess axonal projection density within these major target regions using a machine learning–based segmentation algorithm (see Materials and Methods). We found that VPCNs projected robustly to the BLA, the prefrontal cortex, and, to lesser extent, the lateral habenula and the parasubthalamic nucleus (Fig. 1*B*–*D*). This projection pattern was concordant with general BFCN projections to the prefrontal cortex and the amygdala.

**Figure 2.**
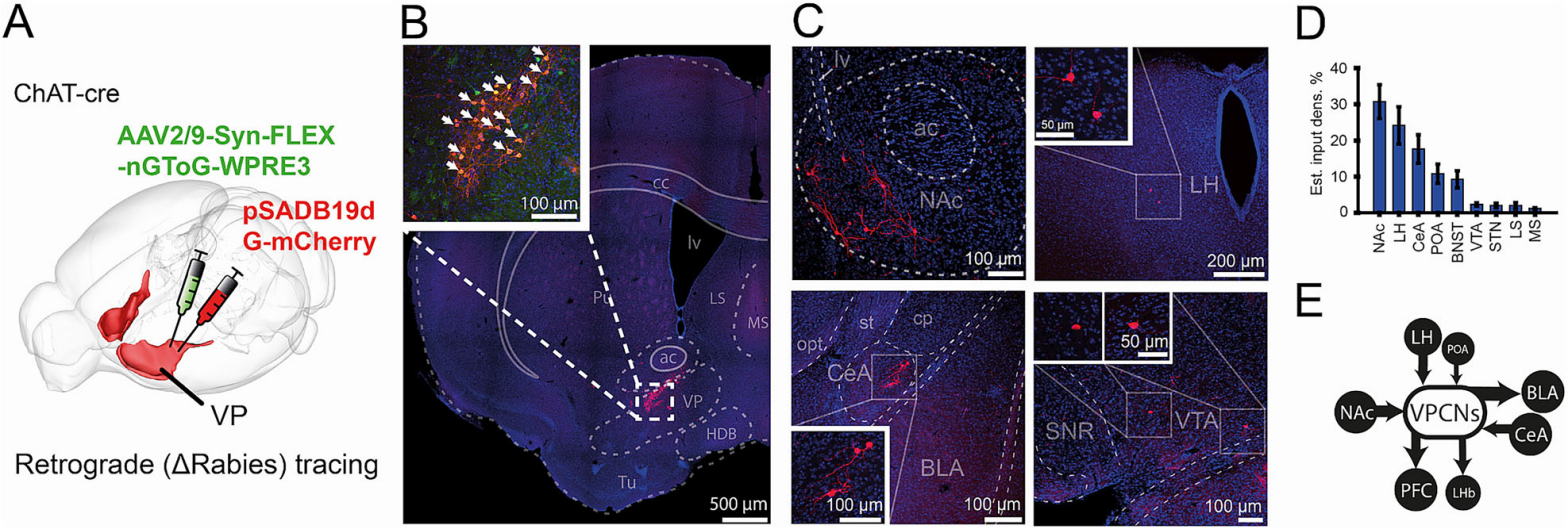
VPCNs receive inputs from the limbic system. ***A***, Schematic illustration of the injection site in the VP of a ChAT-Cre mouse, showing the delivery of the helper virus (green) and pseudotyped rabies virus (red). See reconstructed injection sites in Figure S1*B*. ***B***, Fluorescent image of the injection site. Inset, cells coexpressing the helper and rabies viruses (white arrowheads). ***C***, Fluorescent images showing input cells in the nucleus accumbens (NAc), the lateral hypothalamus (LH), the central amygdala (CeA), and the ventral tegmental area (VTA). ***D***, Estimated input density as a percentage of total input cells (*n* = 703 cells from 6 mice) across various brain regions. Bars and error bars indicate mean ± SEM (NAc, 30.73 ± 4.69%; LH, 24.18 ± 5.14%; CeA, 17.64 ± 3.94%; POA, 10.81 ± 2.67%; BNST, 9.25 ± 2.36%; VTA, 2.28 ± 0.48%; STN, 1.99 ± 0.62%; LS, 1.99 ± 0.79%; MS, 1.14 ± 0.28%). ***E***, Schematic summary of the major input and output regions of the VPCNs.

Next, we performed input mapping of VPCNs by monotranssynaptic rabies tracing (Fig. 2*A*). We found that VPCNs received the majority of their monosynaptic inputs from the nucleus accumbens, the lateral hypothalamus, and the central amygdala, with smaller contributions from the preoptic area and the bed nucleus of stria terminalis (Fig. 2*B*–*E*). This afferent connectivity aligns with previously reported inputs to BFCNs (Do et al., 2016; Hu et al., 2016).

### Ventral pallidal cholinergic neurons resemble the bursting type of BFCNs

**Figure 3.**
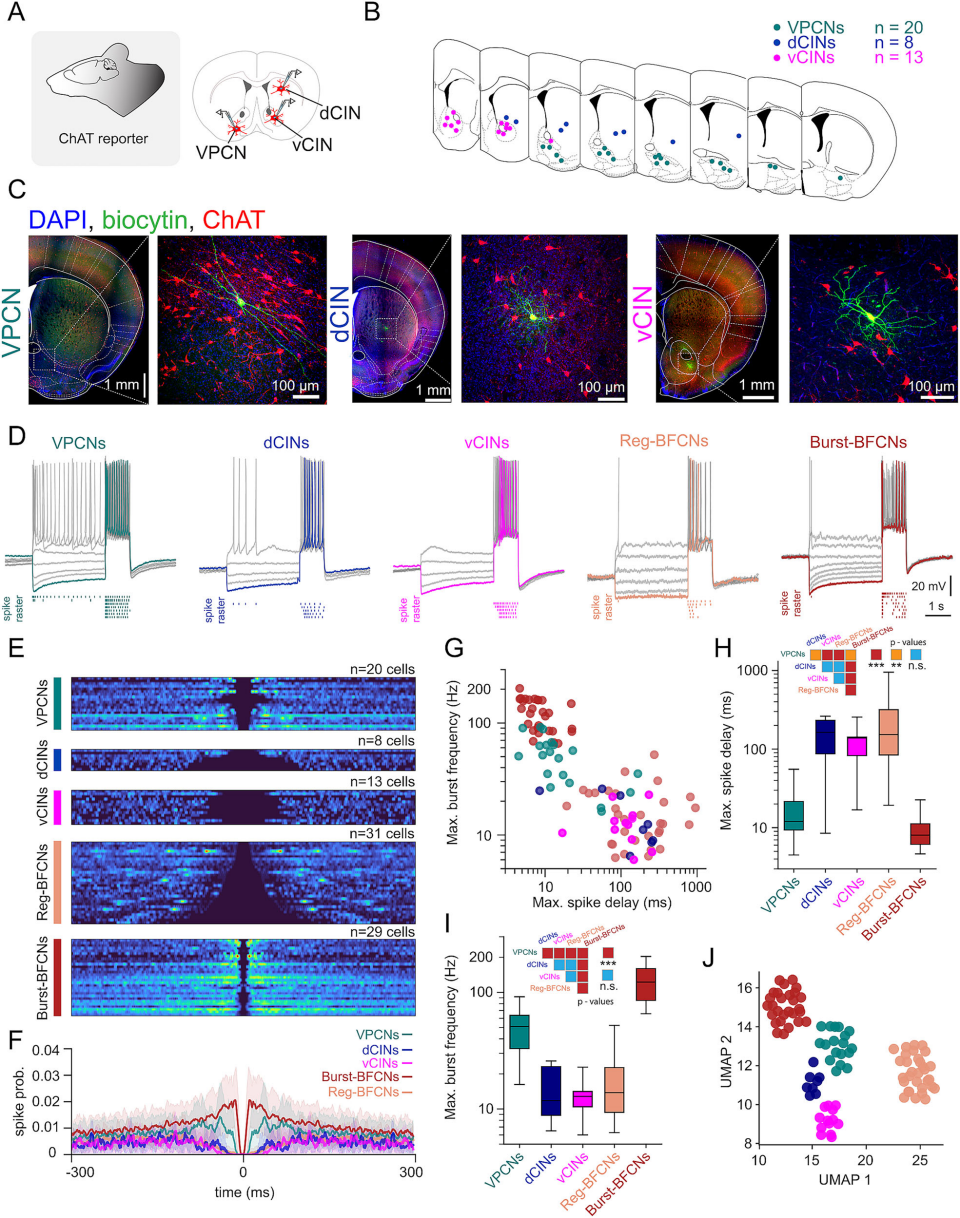
VPCNs resemble burst-firing cholinergic cells of the basal forebrain. ***A***, Schematic of the in vitro acute slice recording experiment. ***B***, Locations of the recorded VPCNs (*n* = 20), dCINs (*n* = 8), and vCINs (*n* = 13). ***C***, Representative confocal images of a recorded and biocytin-filled (green) VPCN (left; scale bars, 1 and 0.1, mm respectively), a dCIN (middle; scale bars, 1 and 0.1 mm, respectively), and a vCIN (right; scale bars, 1 and 0.1 mm, respectively) from a reporter mouse expressing red fluorescent protein in all cholinergic neurons. ***D***, Representative firing patterns of ventral pallidal, dorsal and ventral striatal, and basal forebrain burst- and regular-firing cholinergic cells. VPCNs displayed short spike delays and high-frequency spike clusters in response to positive current injections, resembling burst-firing BFCNs (Burst-BFCNs). In contrast, both dCINs and vCINs exhibited firing patterns similar to regular-firing BFCNs (Reg-BFCNs). ***E***, Spike ACGs during somatic current injection protocols for all recorded cholinergic neurons, grouped by the cell type. ***F***, Average ACGs for VPCNs (teal, *n* = 20), dCINs (blue, *n* = 8), vCINs (magenta, *n* = 13), Burst-BFCNs (red, *n* = 29), and Reg-BFCNs (pink, *n* = 31). Solid lines represent the mean, and shaded regions indicate SEM. ***G***, Maximal burst frequency plotted against maximal spike delay for all recorded cells on a log–log scale, color coded by the cell type. ***H***, Population statistics comparing the maximum spike delay across all cholinergic neuron types. **p < 0.01; ***p < 0.001; Mann–Whitney *U* test. Maximal spike delay, VPCNs versus dCINs, *U* = 23.50; *p* = 0.0044; VPCNs versus Burst-BFCNs, *U* = 436.50; *p* = 0.00298; VPCNs versus Reg-BFCNs, *U* = 41.00; *p* = 2.21 × 10^−7^; dCINs versus Burst-BFCNs, *U* = 221.00; *p* = 0.00012; dCINs versus Reg-BFCNs, *U* = 104.00; *p* = 0.50502; Burst-BFCNs versus Reg-BFCNs, *U* = 896.00; *p* = 2.08 × 10^−11^; VPCNs versus vCINs, *U* = 22.00; *p* = 7.47 × 10^−5^; dCINs versus vCINs, *U* = 58.00; *p* = 0.69; vCINs versus Reg-BFCN, *U* = 165.00; *p* = 0.35; vCINs versus Burst-BFCNs, *U* = 374.00; *p* = 4.80 × 10^−7^. ***I***, Population statistics comparing the maximum burst frequency across all cholinergic neuron types. **p < 0.01; ***p < 0.001; Mann–Whitney *U* test. Maximal burst frequency, VPCNs versus dCINs, *U* = 152.00; *p* = 4.31 × 10^−5^; VPCNs versus Burst-BFCNs, *U* = 29.00; *p* = 1.16 × 10^−7^; VPCNs versus Reg-BFCNs, *U* = 575.00; *p* = 3.34 × 10^−7^; dCINs versus Burst-BFCNs, *U* = 0.00; *p* = 2.021 × 10^−5^; dCINs versus Reg-BFCNs, *U* = 119.00; *p* = 0.88; Burst-BFCNs versus Reg-BFCNs, *U* = 0.00; *p* = 1.54 × 10^−11^. VPCNs versus vCINs, *U* = 256.00; *p* = 3.76 × 10^−6^; dCINs versus vCINs, *U* = 53.00; *p* = 0.97; vCINs versus Reg-BFCN, *U* = 168.00; *p* = 0.40; vCINs versus Burst-BFCNs, *U* = 0.00; *p* = 3.12 × 10^−7^. ***J***, UMAP of a high-dimensional electrophysiological feature set extracted from all cholinergic cells (see Materials and Methods), color coded by the cell type. Please note that UMAP does not preserve global topology or scale but rather emphasizes local neighborhood structure, so clusters may not appear close in the embedding despite being related in the original space (McInnes et al., 2018; Healy and McInnes, 2024).

BFCNs form two distinct cell types, a synchronous population of neurons that fire bursts that correlate with cortical activity (Burst-BFCNs) and a regular rhythmic firing group of cells that synchronizes with cortical activity in a behavior-predictive manner (Reg-BFCN; Laszlovszky et al., 2020; Lozovaya et al., 2024). The firing patterns of striatal cholinergic interneurons (CINs) resemble that of Reg-BFCNs (Inokawa et al., 2010; Zhang et al., 2018; Cox and Witten, 2019). We characterized intrinsic electrophysiological properties of VPCNs, BFCNs, and CINs with identical protocols to determine how VPCN activity is related to the above better-known cholinergic populations. We included two separate striatal populations of dorsal CINs (dCINS) recorded from the dorsal striatum and ventral CINs (vCINS) recorded from the nucleus accumbens (Fig. 3*A*–*C*).

We prepared acute slices from mice expressing fluorescent proteins selectively in cholinergic neurons (see Materials and Methods) and performed whole-cell patch–clamp recordings from *n* = 20 VPCNs. These recordings were contrasted to novel acute slice recordings of dCINs (*n* = 8) and vCINs (*n* = 13) as well as previously obtained traces (Laszlovszky et al., 2020) of Burst-BFCNs and Reg-BFCNs (*n* = 29 and 31, respectively; Fig. 3*D*). Autocorrelations of VPCN activity during somatic current injection protocols revealed a homogeneous bursting phenotype, resembling Burst-BFCNs of the HDB and SI (Fig. 3*E*), markedly different from dCINS, vCINs, and Reg-BFCNs. However, VPCNs were differentiated from Burst-BFCNs by somewhat longer refractory periods and lower maximal burst frequency (Fig. 3*G–I*). In sum, VPCNs form a distinct group based on their intrinsic electrophysiological properties, closely resembling Burst-BFCNs of the HDB and SI (Fig. 3*J*; Fig. S1*C*).

### VPCNs respond to rewards, punishments, and reward-predicting cues

**Figure 4.**
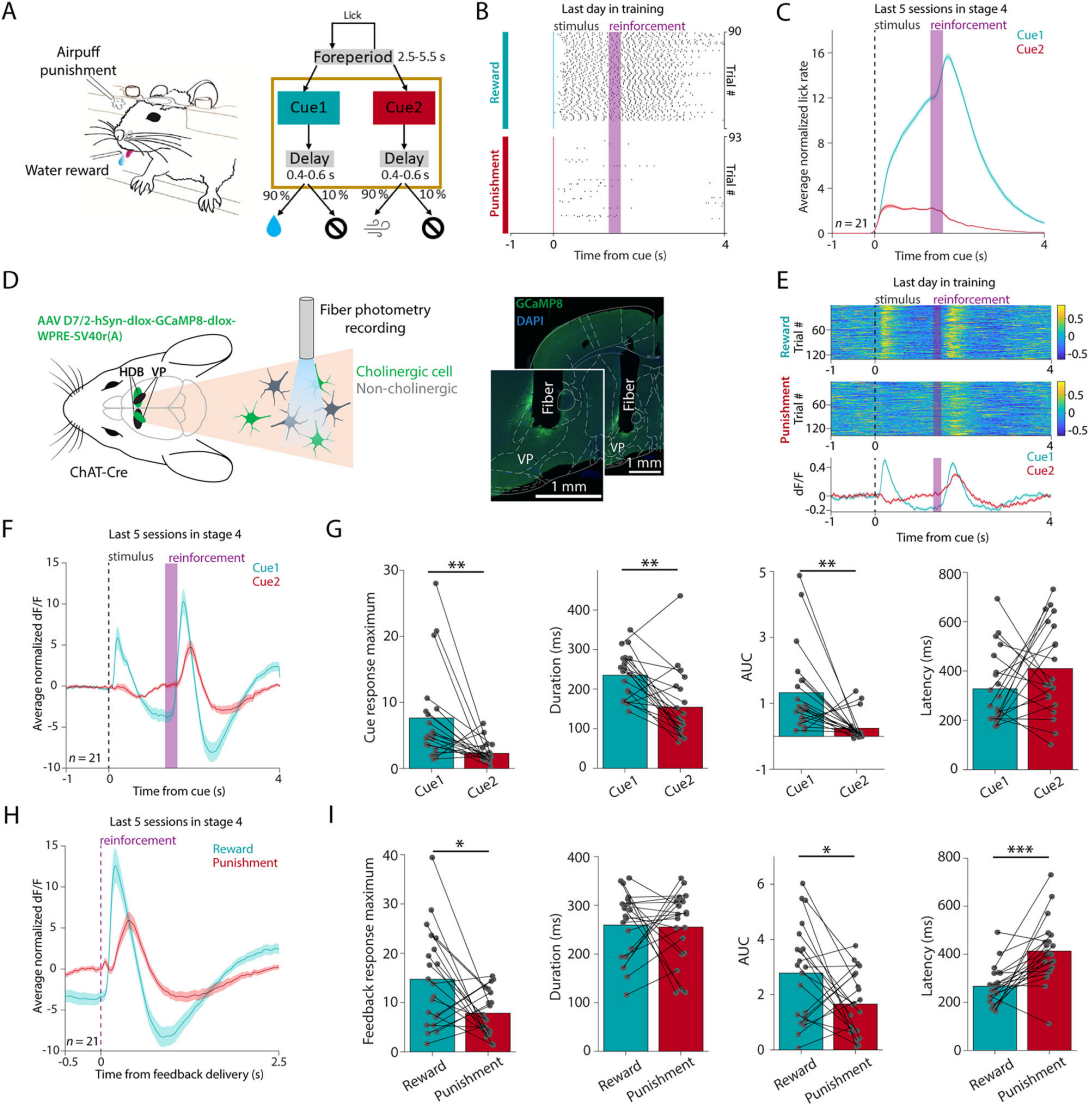
Cholinergic cells in the VP respond differently to the reward- and punishment-predicting cues. ***A***, Schematic of the head-fixed probabilistic pavlovian conditioning task. Created using Mathis (2020), Classical Conditioning Mouse, Zenodo, https://doi.org/10.5281/zenodo.3925907, under Creative Commons 4.0 license (https://creativecommons.org/licenses/by/4.0/). The original image was not modified. ***B***, Raster plot of individual licks aligned to the onset of Cue 1 and Cue 2, respectively, from an example recording session on the last day of training. The mouse showed preferential anticipatory licking to the reward-predicting Cue 1. ***C***, Average *z*-scored anticipatory lick rate of all animals (*n* = 21), aligned to the reward-predicting Cue 1 (green) and the punishment-predicting Cue 2 (red). Error shades indicate SEM. The last five sessions in Stage 4 were used. ***D***, Left, Schematic representation of the fiber photometry measurements. We injected AAV D7/2-hSyn-dlox-GCaMP8-dlox-WPRE-SV40r(A) into the VP and the HDB in the two hemispheres of ChAT-Cre mice and measured cholinergic calcium signals using fiber photometry. Created using Petrucco (2020), Mouse head schema, Zenodo, https://doi.org/10.5281/zenodo.3925902, and Scidraw (2020), Neuron silhouette, Zenodo, https://doi.org/10.5281/zenodo.3925927, under Creative Commons 4.0 license (https://creativecommons.org/licenses/by/4.0/). The original image was not modified. Right, Representative fluorescent histological image of the measurement site (green, GCaMP8; blue, DAPI nuclear staining). Scale bars, 1 mm. ***E***, Example fiber photometry recording of VPCNs from an example recording session on the last day of training. Top, Normalized d*F*/*F* traces of all rewarded and punished trials aligned to cue onset, color coded (blue, low values; yellow, high values). Bottom, Average d*F*/*F* traces from the same session. Error shades indicate SEM. ***F***, Average *z*-scored d*F*/*F* of VPCNs aligned to the reward-predicting Cue 1 (green) and the punishment-predicting Cue 2 (red), averaged across all animals (*n* = 21). Error shades indicate SEM. The last five sessions in Stage 4 were used. ***G***, From left to right, comparison of response magnitude, duration, integral, and latency between VPCN responses to the reward-predicting Cue 1 and the punishment-predicting Cue 2. Each dot represents the session average of a single animal. AUC, area under the curve. Bar graphs show mean. **p < 0.01; maximum, *W* = 27; *p* = 0.0021; integral, *W* = 27; *p* = 0.0021; duration, *W* = 27; *p* = 0.0021; Wilcoxon signed-rank test. ***H***, The same as in panel ***F*** but aligned to reward (green) and punishment delivery (red). Error shades indicate SEM. The last five sessions in Stage 4 were used. ***I***, The same as in panel ***H*** but comparing VPCN responses to reward and punishment. Each dot represents the session average of a single animal. Bar graphs show mean. *p < 0.05; ***p < 0.001; maximum, *W* = 43; *p* = 0.0117; integral, *W* = 54; *p* = 0.0325; latency, *W* = 14; *p* = 0.0004; Wilcoxon signed-rank test.

To determine the behavioral correlates of VPCNs, we trained head-fixed mice on pavlovian conditioning, where two pure tones of different pitch (Cue1 and Cue2) predicted water reward or air-puff punishment, respectively (Fig. 4*A*). Mice learned these task contingencies, indicated by preferential anticipatory licking after the reward-predicting tone (Fig. 4*B*,*C*; Fig. S2*A*) and a conditioned squinting response after the tone predicting air-puff punishment (Fig. S2*C*–*G*).

ChAT-Cre mice (*n* = 22) were injected with AAV D7/2-hSyn-dlox-GCaMP8-dlox-WPRE-SV40r(A) to express the fluorescent calcium indicator GCaMP8 in VPCNs and implanted with optic fibers in the VP and HDB regions on the two sides (Fig. 4*D*). We performed fiber photometry recordings of bulk calcium levels of cholinergic neurons, while mice performed the pavlovian task. We found that both VPCNs and HDB cholinergic neurons (HDBCNs) consistently responded to cues, rewards, and punishments (Fig. 4*E–G*; Fig. S3). While the reward-predicting Cue1 evoked large increases of calcium in VPCNs, the punishment-predicting Cue2 induced smaller and more variable responses (Fig. 4*F*). Nevertheless, most mice showed a detectable peak response after Cue 2 as well, allowing further quantification (*n* = 16 of 21 mice tested; *W* < 88,276; *p* < 0.05; Wilcoxon signed-rank test; Fig. S4*A*–*C*). Overall, responses to Cue1 were significantly larger in amplitude and integral and longer in duration (Fig. 4*G*; including mice with individually significant Cue 2 response, Fig. S4*D*).

Both rewards and punishments evoked consistent, large increases in VPCN calcium signals. A quantitative comparison revealed that reward responses were larger and faster than punishment responses (Fig. 4*H*,*I*).

**Figure 5.**
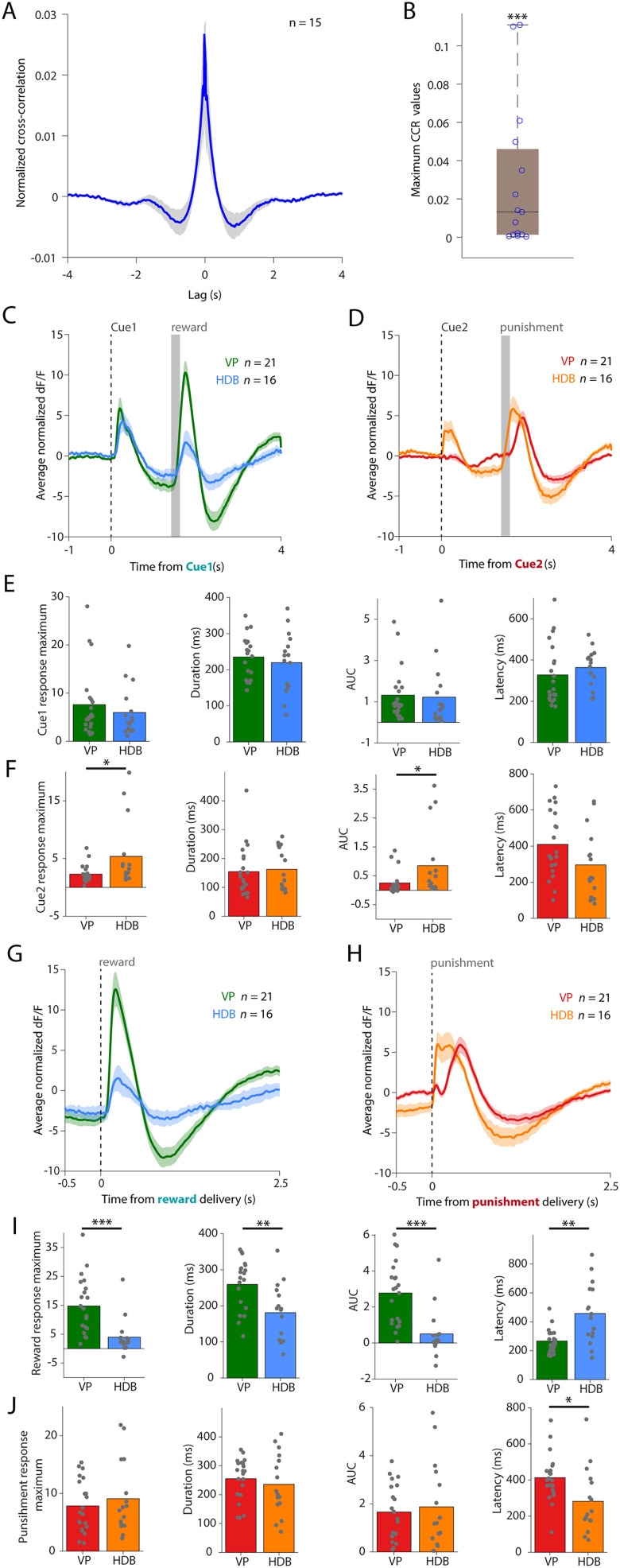
Differences of cholinergic reward and punishment responses between VP and HDB. ***A***, CCR of HDBCN and VPCN bulk calcium recordings, averaged across all animals (*n* = 15 mice with both signals accepted; see Materials and Methods). ***B***, Maximal CCR values averaged per mice (*n* = 15). ***p < 0.001 for CCR > 0; *W* = 0.00; *p* = 0.0001; Wilcoxon signed-rank test. ***C***, Average *z*-scored d*F*/*F* of VPCNs (green) and HDBCNs (blue) aligned to the reward-predicting cues, averaged across all animals (VP, *n* = 21; HDB, *n* = 16). Error shades indicate SEM. ***D***, Average *z*-scored d*F*/*F* of VPCNs (red) and HDBCNs (orange) aligned to the punishment-predicting cues, averaged across all animals (VP, *n* = 21; HDB, *n* = 16). Error shades indicate SEM. ***E***, From left to right, comparison of Cue1 response magnitude, duration, integral, and latency between VPCNs (*n* = 21) and HDBCNs (*n* = 16). Each dot represents the session average of a single animal. Bar graphs show mean. Mann–Whitney *U* test. ***F***, From left to right, comparison of Cue2 response magnitude, duration, integral, and latency between VPCNs (*n* = 21) and HDBCNs (*n* = 16). Each dot represents the session average of a single animal. Bar graphs show mean. *p < 0.05; maximum, *U* = 85; *p* = 0.0114; integral, *U* = 97; *p* = 0.0307; Mann–Whitney *U* test. ***G***, Average *z*-scored d*F*/*F* of VPCNs (green) and HDBCNs (blue) aligned to rewards, averaged across all animals (VP, *n* = 21; HDB, *n* = 16). Error shades indicate SEM. ***H***, Average *z*-scored d*F*/*F* of VPCNs (red) and HDBCNs (orange) aligned to punishments, averaged across all animals (VP, *n* = 21; HDB, *n* = 16). Error shades indicate SEM. ***I***, From left to right, comparison of reward response magnitude, duration, integral, and latency between VPCNs (*n* = 21) and HDBCNs (*n* = 16). Each dot represents the session average of a single animal. Bar graphs show mean. **p < 0.01; ***p < 0.001; maximum, *U* = 43; *p* = 0.0001; duration, *U* = 73; *p* = 0.0038; integral, *U* = 41; *p* = 0.0001; latency, *U* = 69; *p* = 0.0025; Mann–Whitney *U* test. ***J***, From left to right, comparison of punishment response magnitude, duration, integral. and latency between VPCNs (*n* = 21) and HDBCNs (*n* = 16). Each dot represents the session average of a single animal. Bar graphs show mean. *p < 0.05; latency, *U* = 86; *p* = 0.0125; Mann–Whitney *U* test.

Next, we directly compared VPCN calcium responses with parallel recordings from the HDB of the basal forebrain (Fig. 5). We found that the response patterns were qualitatively similar, including cue, reward, and punishment responses (Hegedüs et al., 2023). These signal correlations were accompanied by consistent positive moment-by-moment noise correlations revealed by CCR analysis, showing a zero-lag–positive correlation flanked by negative correlations around a characteristic delay of ∼0.8 s (−0.721 and 0.861 s; Fig. 5*A*,*B*). This indicates an ongoing coordination of cholinergic neurons of the two regions, likely caused by common excitatory inputs. However, a quantitative comparison uncovered notable differences as well: while responses to the reward-predicting Cue1 were almost identical (Fig. 5*C*,*E*), VPCNs exhibited smaller responses to the punishment-predicting Cue2 (Fig. 5*D*,*F*; Fig. S4*E*). While reward responses were much larger, longer, and faster in VP (Fig. 5*G*,*I*), the punishment responses were comparable in amplitude but slower in the VP (Fig. 5*H*,*J*). These results revealed qualitatively similar response patterns in cholinergic neurons of the VP and the HDB but also a quantitative preference to appetitive stimuli in VPCNs.

### Most putative VPCNs show spike responses to salient stimuli

**Figure 6.**
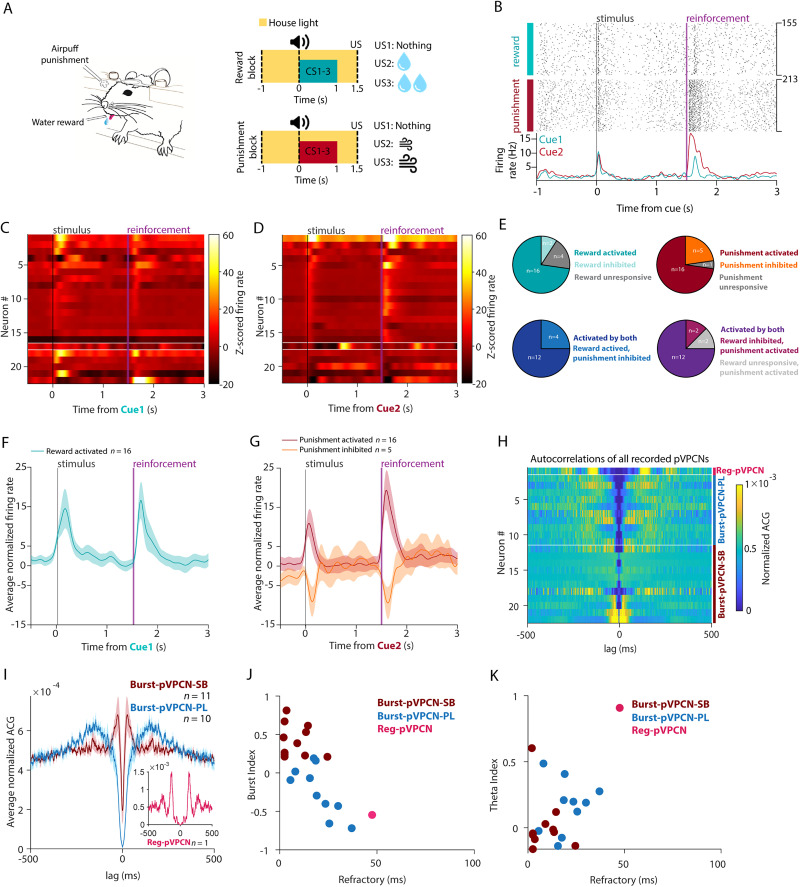
Most putative VPCNs show spike responses to salient stimuli. ***A***, Schematic of the pavlovian conditioning task. ***B***, Top, Raster plot of spike times aligned to cue onset of an example pVPCN during the pavlovian task in rewarded and punished trials. Bottom, Corresponding PETHs (green, rewarded trials; red, punished trials). ***C***, *Z*-scored PETHs of all recorded pVPCNs (*n* = 22) during rewarded trials shown on a heatmap, sorted by the punishment response magnitudes (for consistent ordering across panels ***C*** and ***D***). Cells activated significantly after punishment are shown above the top white line, while those that were significantly inhibited by punishment are shown below the bottom white line. ***D***, *Z*-scored PETHs of all recorded pVPCNs (*n* = 22) during punishment trials shown as a heatmap. Cells activated significantly after punishment are shown above the top white line, while those that were significantly inhibited are shown below the bottom white line. ***E***, Pie charts showing the proportions of pVPCN response types. Top left, Reward responses of all recorded pVPCNs (*n* = 22). Top right, Punishment responses of all pVPCNs. Bottom left, Punishment responses of reward-activated pVPCNs (*n* = 16). Bottom right, Reward responses of punishment-activated pVPCNs (*n* = 16). ***F***, The average normalized firing rate of reward-activated pVPCNs (*n* = 16) aligned to cue onset during rewarded trials (mean ± SEM). ***G***, The average normalized firing rate of punishment-activated pVPCNs (*n* = 16, dark red) and punishment-inhibited pVPCNs (*n* = 5, light red) aligned to cue onset during punishment trials (mean ± SEM). ***H***, Autocorrelations (ACG, normalized to a sum of one) of all recorded pVPCNs (*n* = 22). Reg-pVPCN, above the top white line; Burst-pVPCN-PLs (*n* = 10), between the two horizontal white lines; Burst-pVPCN-SBs (*n* = 11), below the bottom white line; sorted by Burst Index within each group. ***I***, Normalized autocorrelation of all recorded pVPCNs averaged by the firing pattern type. Dark red, Burst-pVPCN-SB (*n* = 11); blue, Burst-pVPCN-PL (*n* = 10); light red in inset, Reg-pVPCN (*n* = 1). ***J***, Burst Index versus refractory period of pVPCNs, color coded by the firing pattern type. ***K***, Theta Index versus refractory period of pVPCNs, color coded by the firing pattern type.

To assess the spiking heterogeneity of VPCNs corresponding to these bulk calcium responses, we analyzed the activity of putative VPCNs (pVPCNs) recorded in a similar pavlovian conditioning task. In this task, different auditory cues predicted large water reward, small reward, or no reward, large air-puff punishment, small punishment, or no punishment in blocks of positive and negative valence trials (Stephenson-Jones et al., 2020; Fig. 6*A*). It was demonstrated that VP neurons (*n* = 331 from 6 mice) fell into four distinct response categories by hierarchical clustering of the first three principal components of the *Z*-scored neuronal responses to reward and punishment [Stephenson-Jones et al. (2020), their Fig. 1], and optogenetic tagging of glutamatergic and GABAergic neurons unambiguously identified two clusters as GABAergic and one as glutamatergic. The remaining “Type 1” neurons (*n* = 22/331) did not contain any glutamatergic or GABAergic neurons and were therefore identified as pVPCNs [Stephenson-Jones et al. (2020), their Fig. S1].

We found that most pVPCNs showed precise reward and punishment responses similar to what was shown for BFCNs in multiple nuclei (Hangya et al., 2015; Laszlovszky et al., 2020; Hegedüs et al., 2023; Fig. 6*B*–*D*). Most pVPCNs were activated by rewards, punishments, and conditioned stimuli, while a smaller population showed activation by positive valence and inhibition by negative valence stimuli (Fig. 6*C*–*G*). These responses were unlike those described for CINs, especially regarding the well-characterized “pause-burst” reward responses of CINs in dorsal striatum (Inokawa et al., 2010; Zhang et al., 2018; Cox and Witten, 2019). Nevertheless, a few pVPCNs showed more delayed and sustained reward-elicited firing rate increase resembling those of CINs (Fig. S5*A*,*B*). These two types could even be recorded concurrently on the same electrode, suggesting that they are spatially mixed.

We also examined the autocorrelation of pVPCNs. Consistent with our in vitro recording, most pVPCNs showed a bursting in vivo firing pattern with a refractory period that was somewhat longer than what was previously shown for Burst-BFCNs (Fig. 6*H*,*I*; Laszlovszky et al., 2020). Indeed, when we categorized pVPCNs to strongly bursting Burst-pVPCNs (Burst-pVPCN-SB), Poisson-like Burst-pVPCNs (Burst-pVPCN-PL), and regular rhythmic pVPCNs (Reg-pVPCNs) based on their burstiness and refractory period as was done for BFCNs (Laszlovszky et al., 2020), we found *n* = 11/22 Burst-pVPCN-SB and *n* = 10/22 Burst-pVPCN-PL neurons, a firing pattern distribution resembling cholinergic neurons of the HDB (Fig. 6*J*,*K*). Corroborating that a small fraction of VPCNs might be CIN-like, one pVPCN showed long refractory and theta-rhythmicity characteristic of both CINs and Reg-BFCNs (*n* = 1/22 Reg-pVPCN).

### Pupil size correlates with VPCN activity

**Figure 7.**
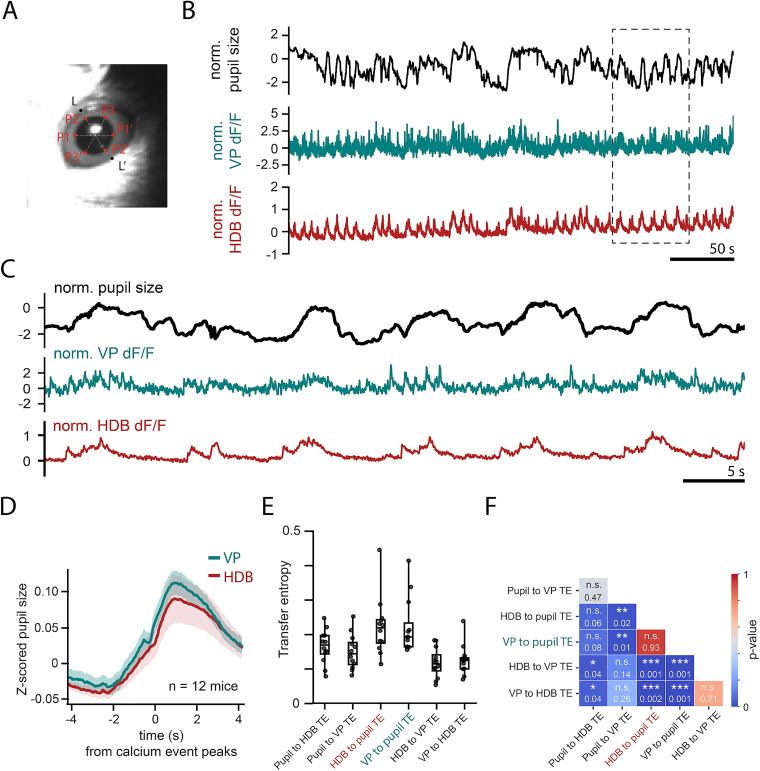
The pupil size correlates with VPCN activity. ***A***, Representative image from a video recording synchronized with fiber photometry measurements of VP and HDBCN activity. The pupil size was tracked using DeepLabCut, trained to identify pupil edges (P1–P3, P1’–P3’) and eyelid positions (L1–L1’). ***B***, Representative traces showing normalized pupil size (black) and normalized cholinergic activity in the VP (teal) and HDB (red). The boxed region is expanded in panel ***C***. ***C***, Magnified view of the boxed region in panel ***B***, illustrating that both VP and HDB cholinergic activity peaks are strongly synchronized with periods of pupil dilation. ***D***, The average pupil size triggered by transient calcium peaks of VPCNs and HDBCNs (*n* = 12 mice). ***E***, Population statistics comparing transfer entropy, which quantifies directional information flow between the pupil size and calcium activity of VPCNs and HDBCNs. ***F***, Significance matrix for the TE analysis shown in panel ***E***. Note that TE values from HDB or VP to pupil are not significantly different (*U* = 74.0; *p* = 0.93; Mann–Whitney *U* test).

Changes in pupil diameter under constant illumination were shown to be predicted by cholinergic transients originating from the basal forebrain (Nelson and Mooney, 2016; Reimer et al., 2016; Jing et al., 2020; Neyhart et al., 2024). We tested whether VPCN activity correlated with changes in pupil diameter as well by monitoring pupil diameter in parallel with VPCN and HDBCN bulk calcium signals (*n* = 12 mice; Fig. 7*A*–*C*).

As expected, pupil dilations were temporally predicted by calcium transients recorded in HDBCNs of the basal forebrain. Similarly, we found that VPCN calcium peaks showed a comparable level of correlation with forthcoming pupil dilations (Fig. 7*B*–*D*). To perform a quantitative comparison of the predictive value of VPCN and HDBCN signals, we calculated TE, an information theory measure of predictability across time series that is not restricted to the linear domain (Gourévitch and Eggermont, 2007; Fig. 7*E*). As expected based on the above temporal dynamics, prediction of the pupil size based on cholinergic signals (HDB to pupil TE and VP to pupil TE) were characterized by the highest TE values. At the same time, VPCNs showed comparable predictive values in terms of the pupil size as the HDBCNs (Fig. 7*F*). These results revealed that cholinergic neurons of the VP showed correlations with pupil dynamics.

## Discussion

We demonstrated that most VPCNs belong to the basal forebrain cholinergic projection system based on their hodology, intrinsic biophysical properties, and in vivo physiological responses to behaviorally salient appetitive and aversive events.

The mediodorsal thalamus is considered a primary output of VP, along with parts of the reticular and paraventricular thalamic regions (Zahm et al., 1996; Tripathi et al., 2013; Root et al., 2015). The VP also sends important projections to the lateral habenula and the VTA, which were shown to express PV and contain both GABAergic and glutamatergic components, linked to different aspects of depression (Knowland et al., 2017). Additionally, the VP sends topographically organized projections to the lateral hypothalamus and GABAergic efferents to the subthalamic nucleus and projects back robustly to the nucleus accumbens, its major source of afferents (Root et al., 2015; Soares-Cunha et al., 2022; Domingues et al., 2023). While most of these projections are considered GABAergic, a strong cholinergic component of the VP to BLA pathway has been described (Root et al., 2015; Kim et al., 2024), while a cholinergic cortical projection was also assumed (Zaborszky et al., 2012). Concerning BFCNs, Do and colleagues characterized whole-brain distribution of axonal projections and found the hippocampus, piriform area, ventral striatum, amygdala, and neocortical regions as main BFCN targets, though these were not stratified according to input cell location within the BF (Do et al., 2016). Except for an absence of hippocampal targets that are known to receive their cholinergic input from rostral BF (Agostinelli et al., 2019), we found VPCN projections consistent with BFCN outputs.

The densest input to VP is provided by GABAergic fibers from the nucleus accumbens, complemented by VTA/SNc dopaminergic, dorsal raphe serotonergic, STN glutamatergic, infralimbic cortical, and amygdalar afferents (Root et al., 2015). It has been shown that besides dopaminergic, the VTA also provides GABAergic and glutamatergic VP inputs (Hnasko et al., 2012; Root et al., 2015). Whole-brain monosynaptic inputs to BFCNs were described by Hu et al. (2016), pointing to the caudoputamen, central amygdala, lateral hypothalamus, nucleus accumbens, and VTA as major sources of afferents, in line with earlier reports (Záborszky and Cullinan, 1992). We found that VPCNs received most of their monosynaptic inputs from nucleus accumbens, lateral hypothalamus, and central amygdala, consistently with BFCNs in general. Although whole-brain studies identified some cortical input sources to BFCNs (Do et al., 2016; Hu et al., 2016), the classical view holds that most cortical basal forebrain inputs are from the prefrontal cortex arriving onto GABAergic BF neurons; thus BFCNs only receive indirect cortical inputs via local inhibitory cells (Gaykema and Zaborszky, 1997; Zaborszky et al., 1997, 2012). In line with the latter, we did not identify direct cortical inputs to VPCNs. In summary, input–output mapping of VPCNs suggests that they are full-fledged members of the basal forebrain cholinergic projection system.

BFCNs were shown to be either early or late firing in acute slice experiments (Unal et al., 2012) and later demonstrated to form two distinct types of regular rhythmic and bursting neurons (Khateb et al., 1992; Alonso et al., 1996; Szymusiak et al., 2000) both in the nucleus basalis and in the HDB in vivo (Laszlovszky et al., 2020). Striatal CINs resemble Reg-BFCNs regarding their firing patterns in their slow-theta rhythmicity and long functional refractory period (Inokawa et al., 2010; Laszlovszky et al., 2020). We found that most VPCNs in vitro as well as pVPCNs in vivo showed bursting properties like Burst-BFCNs, with a few exceptions that fired like Reg-BFCNs and CINs. Of note, VPCNs showed slightly but distinctively longer refractory periods in their ACGs than BFCNs, the significance of which should be determined by future studies. These results suggest that most VPCNs fire in accordance with a topographical anteroposterior gradient of bursting cholinergic neurons within the basal forebrain (Laszlovszky et al., 2020).

VPCNs responded to rewards, punishments, and reward-predicting stimuli, consistent with both BFCN (Lovett-Barron et al., 2014; Hangya et al., 2015; Harrison et al., 2016; Sturgill et al., 2020; Robert et al., 2021; Allard and Hussain Shuler, 2023; Hegedüs et al., 2023) and VP function in reward coding, motivation, and associative learning (Tindell, 2004; Smith et al., 2009; Wassum et al., 2009; Richard et al., 2016, 2018; Saga et al., 2017; Wulff et al., 2019; Stephenson-Jones et al., 2020; Ottenheimer et al., 2020a; Hegedüs et al., 2021; Soares-Cunha et al., 2022). When we performed a direct comparison with the HDB nucleus of the BF in pavlovian conditioning, we found that VPCN and HDBCN calcium signals were robustly positively correlated. Nevertheless, bulk calcium recordings also revealed a bias in VPCNs toward reward responses, with faster and larger calcium responses to rewards but slower responses to punishments. This is in line with the known importance of VP in the reward aspects of learning (Tindell et al., 2006; Smith et al., 2009; Prasad et al., 2020), recent findings on the role of HDB in aversive coding, and learning from negative experience (Hangya et al., 2015; Hegedüs et al., 2024) and supports the conclusion of Ottenheimer et al. that the VP processes certain aspects of reward independently of the nucleus accumbens, based on faster and more robust reward responses in the VP (Ottenheimer et al., 2018). Indeed, our results suggest that faster-than-striatal reward responses in the VP may arrive through VPCNs.

Spike responses of most pVPCNs to rewards, punishments, and conditioned stimuli showed temporal dynamics characteristic to other BFCNs (Hangya et al., 2015; Sturgill et al., 2020; Hegedüs et al., 2023), while a few neurons exhibited striatal-like pause–burst responses to rewards (Inokawa et al., 2010). Most VPCNs showed correlated responses to positive and negative valence stimuli (Stephenson-Jones et al., 2020) similar to BFCNs (Hangya et al., 2015; Sturgill et al., 2020; Hegedüs et al., 2023), while a few VPCNs exhibited strong bias toward positive or negative outcomes, in line with a recent study that used olfactory stimuli (Kim et al., 2024). These results suggest an involvement of VPCNs in explicit learning likely including fear learning (Akmese et al., 2023; Ji et al., 2023), similar to what was shown for BFCNs in general (Letzkus et al., 2011; Jiang et al., 2016).

The bias toward rewarding stimuli seen in the bulk calcium recordings was not obvious for the average spiking response of pVPCNs, which could be due to a number of reasons. First, there was a larger fraction of punishment-inhibited than reward-inhibited neurons (23 vs 9%) that may have affected the cell-type–averaged bulk calcium signal. Second, fast spiking responses might have been low-pass filtered by slower biophysical processes, including the dynamics of neurotransmitter spillover and fluorescent dye kinetics. Third, while the reward magnitude was comparable across experiments, the more aversive strong air puffs in the electrophysiology experiments could be a contributor to the larger punishment responses at the individual cellular level. Fourth, we cannot fully rule out that small differences of recording locations within the VP contributed to the differences.

Changes in the pupil size under constant illumination has been linked to multiple neuromodulatory systems (Larsen and Waters, 2018) including noradrenergic (Reimer et al., 2016; de Gee et al., 2017; Bang et al., 2023), cholinergic (Nelson and Mooney, 2016; Reimer et al., 2016; Jing et al., 2020; Neyhart et al., 2024), and serotonergic (Cazettes et al., 2021) activity and were recently shown to reflect learning (Lee and Margolis, 2016) and consolidation processes (Chang et al., 2025) of associative memories. A difference in the time lag between acetylcholine rise and pupil dilation across different cortical areas suggested that different parts of the cholinergic system may have distinct temporal correlations with the pupil size (Neyhart et al., 2024). We tested this by correlating VPCN and HDBCN activity with pupil diameter and found comparable predictive value of the two cholinergic signals in forecasting pupil dilations.

## References

[B1] Agostinelli LJ, Geerling JC, Scammell TE (2019) Basal forebrain subcortical projections. Brain Struct Funct 224:1097–1117. 10.1007/s00429-018-01820-6 30612231 PMC6500474

[B2] Ahrens AM, Meyer PJ, Ferguson LM, Robinson TE, Aldridge JW (2016) Neural activity in the ventral pallidum encodes variation in the incentive value of a reward cue. J Neurosci 36:7957–7970. 10.1523/JNEUROSCI.0736-16.2016 27466340 PMC4961780

[B3] Ahrens AM, Ferguson LM, Robinson TE, Aldridge JW (2018) Dynamic encoding of incentive salience in the ventral pallidum: dependence on the form of the reward cue. eNeuro 5:ENEURO.0328-17.2018. 10.1523/ENEURO.0328-17.2018 PMC5938716 29740595

[B4] Akmese C, Sevinc C, Halim S, Unal G (2023) Differential role of GABAergic and cholinergic ventral pallidal neurons in behavioral despair, conditioned fear memory and active coping. Prog Neuropsychopharmacol Biol Psychiatry 125:110760. 10.1016/j.pnpbp.2023.110760 37031946

[B5] Allard S, Hussain Shuler MG (2023) Cholinergic reinforcement signaling is impaired by amyloidosis prior to its synaptic loss. J Neurosci 43:6988–7005. 10.1523/JNEUROSCI.0967-23.2023 37648452 PMC10586537

[B6] Alonso A, Khateb A, Fort P, Jones BE, Mühlethaler M (1996) Differential oscillatory properties of cholinergic and non-cholinergic nucleus basalis neurons in Guinea pig brain slice. Eur J Neurosci 8:169–182. 10.1111/j.1460-9568.1996.tb01178.x 8713461

[B7] Avila I, Lin S-C (2014) Distinct neuronal populations in the basal forebrain encode motivational salience and movement. Front Behav Neurosci 8:421. 10.3389/fnbeh.2014.00421 25538586 PMC4255619

[B8] Bang D, et al. (2023) Noradrenaline tracks emotional modulation of attention in human amygdala. Curr Biol 33:5003–5010.e6. 10.1016/j.cub.2023.09.074 37875110 PMC10957395

[B9] Berg S, et al. (2019) Ilastik: interactive machine learning for (bio)image analysis. Nat Methods 16:1226–1232. 10.1038/s41592-019-0582-9 31570887

[B10] Cazettes F, Reato D, Morais JP, Renart A, Mainen ZF (2021) Phasic activation of dorsal raphe serotonergic neurons increases pupil size. Curr Biol 31:192–197.e4. 10.1016/j.cub.2020.09.090 33186549 PMC7808753

[B11] Chang H, Tang W, Wulf AM, Nyasulu T, Wolf ME, Fernandez-Ruiz A, Oliva A (2025) Sleep microstructure organizes memory replay. Nature 637:1161–1169. 10.1038/s41586-024-08340-w 39743590 PMC12107872

[B12] Cohen JY, Haesler S, Vong L, Lowell BB, Uchida N (2012) Neuron-type-specific signals for reward and punishment in the ventral tegmental area. Nature 482:85–88. 10.1038/nature10754 22258508 PMC3271183

[B13] Courtin J, Chaudun F, Rozeske RR, Karalis N, Gonzalez-Campo C, Wurtz H, Abdi A, Baufreton J, Bienvenu TCM, Herry C (2013) Prefrontal parvalbumin interneurons shape neuronal activity to drive fear expression. Nature 505:92–96. 10.1038/nature12755 24256726

[B14] Cox J, Witten IB (2019) Striatal circuits for reward learning and decision-making. Nat Rev Neurosci 20:482–494. 10.1038/s41583-019-0189-2 31171839 PMC7231228

[B15] Creed M, Ntamati NR, Chandra R, Lobo MK, Lüscher C (2016) Convergence of reinforcing and anhedonic cocaine effects in the ventral pallidum. Neuron 92:214–226. 10.1016/j.neuron.2016.09.001 27667004 PMC8480039

[B16] de Gee JW, Colizoli O, Kloosterman NA, Knapen T, Nieuwenhuis S, Donner TH (2017) Dynamic modulation of decision biases by brainstem arousal systems. Elife 6:1–36. 10.7554/eLife.23232 PMC5409827 28383284

[B17] Do JP, et al. (2016) Cell type-specific long-range connections of basal forebrain circuit. Elife 5:1–17. 10.7554/eLife.13214 PMC5095704 27642784

[B18] Domingues AV, Rodrigues AJ, Soares-Cunha C (2023) A novel perspective on the role of nucleus accumbens neurons in encoding associative learning. FEBS Lett 597:2601–2610. 10.1002/1873-3468.14727 37643893

[B19] Espinosa N, Alonso A, Lara-Vasquez A, Fuentealba P (2019) Basal forebrain somatostatin cells differentially regulate local gamma oscillations and functionally segregate motor and cognitive circuits. Sci Rep 9:1–12. 10.1038/s41598-019-39203-4 30796293 PMC6384953

[B20] Faget L, Zell V, Souter E, McPherson A, Ressler R, Gutierrez-Reed N, Yoo JH, Dulcis D, Hnasko TS (2018) Opponent control of behavioral reinforcement by inhibitory and excitatory projections from the ventral pallidum. Nat Commun 9:849. 10.1038/s41467-018-03125-y 29487284 PMC5829073

[B21] Farrell MR, Esteban JSD, Faget L, Floresco SB, Hnasko TS, Mahler SV (2021) Ventral pallidum GABA neurons mediate motivation underlying risky choice. J Neurosci 41:4500–4513. 10.1523/JNEUROSCI.2039-20.2021 33837052 PMC8152612

[B22] Fujimoto A, Hori Y, Nagai Y, Kikuchi E, Oyama K, Suhara T, Minamimoto T (2019) Signaling incentive and drive in the primate ventral pallidum for motivational control of goal-directed action. J Neurosci 39:1793–1804. 10.1523/JNEUROSCI.2399-18.2018 30626695 PMC6407294

[B23] Gaykema RP, Zaborszky L (1997) Parvalbumin-containing neurons in the basal forebrain receive direct input from the substantia nigra-ventral tegmental area. Brain Res 747:173–179. 10.1016/S0006-8993(96)01309-1 9042545

[B24] Gourévitch B, Eggermont JJ (2007) Evaluating information transfer between auditory cortical neurons. J Neurophysiol 97:2533–2543. 10.1152/jn.01106.2006 17202243

[B25] Hangya B, Ranade SP, Lorenc M, Kepecs A (2015) Central cholinergic neurons are rapidly recruited by reinforcement feedback. Cell 162:1155–1168. 10.1016/j.cell.2015.07.057 26317475 PMC4833212

[B26] Harrison TC, Pinto L, Brock JR, Dan Y (2016) Calcium imaging of basal forebrain activity during innate and learned behaviors. Front Neural Circuits 10:1–12. 10.3389/fncir.2016.00036 27242444 PMC4863728

[B27] Healy J, McInnes L (2024) Uniform manifold approximation and projection. Nat Rev Methods Primers 4:82. 10.1038/s43586-024-00363-x

[B28] Hegedüs P, Heckenast J, Hangya B (2021) Differential recruitment of ventral pallidal e-types by behaviorally salient stimuli during Pavlovian conditioning. iScience 24:102377. 10.1016/j.isci.2021.102377 33912818 PMC8066429

[B29] Hegedüs P, Sviatkó K, Király B, Martínez-Bellver S, Hangya B (2023) Cholinergic activity reflects reward expectations and predicts behavioral responses. iScience 26:105814. 10.1016/j.isci.2022.105814 36636356 PMC9830220

[B30] Hegedüs P, Király B, Schlingloff D, Lyakhova V, Velencei A, Szabó Í, Mayer MI, Zelenak Z, Nyiri G, Hangya B (2024) Parvalbumin-expressing basal forebrain neurons mediate learning from negative experience. Nat Commun 15:4768. 10.1038/s41467-024-48755-7 38849336 PMC11161511

[B31] Heinsbroek JA, Bobadilla AC, Dereschewitz E, Assali A, Chalhoub RM, Cowan CW, Kalivas PW (2020) Opposing regulation of cocaine seeking by glutamate and GABA neurons in the ventral pallidum. Cell Rep 30:2018–2027.e3. 10.1016/j.celrep.2020.01.023 32049028 PMC7045305

[B32] Hernandez-Jaramillo A, Illescas-Huerta E, Sotres-Bayon F (2024) Ventral pallidum and amygdala cooperate to restrain reward approach under threat. J Neurosci 44:e2327232024. 10.1523/JNEUROSCI.2327-23.2024 38631914 PMC11154850

[B33] Hnasko TS, Hjelmstad GO, Fields HL, Edwards RH (2012) Ventral tegmental area glutamate neurons: electrophysiological properties and projections. J Neurosci 32:15076–15085. 10.1523/JNEUROSCI.3128-12.2012 23100428 PMC3685320

[B34] Hu R, Jin S, He X, Xu F, Hu J (2016) Whole-brain monosynaptic afferent inputs to basal forebrain cholinergic system. Front Neuroanat 10:98. 10.3389/fnana.2016.00098 27777554 PMC5056182

[B35] Inokawa H, Yamada H, Matsumoto N, Muranishi M, Kimura M (2010) Juxtacellular labeling of tonically active neurons and phasically active neurons in the rat striatum. Neuroscience 168:395–404. 10.1016/j.neuroscience.2010.03.062 20371269

[B36] Ito M, Doya K (2009) Validation of decision-making models and analysis of decision variables in the rat basal ganglia. J Neurosci 29:9861–9874. 10.1523/JNEUROSCI.6157-08.2009 19657038 PMC6666589

[B37] Ji Y-W, et al. (2023) Plasticity in ventral pallidal cholinergic neuron-derived circuits contributes to comorbid chronic pain-like and depression-like behaviour in male mice. Nat Commun 14:2182. 10.1038/s41467-023-37968-x 37069246 PMC10110548

[B38] Jiang L, Kundu S, Lederman JD, López-Hernández GY, Ballinger EC, Wang S, Talmage DA, Role LW (2016) Cholinergic signaling controls conditioned fear behaviors and enhances plasticity of cortical-amygdala circuits. Neuron 90:1057–1070. 10.1016/j.neuron.2016.04.028 27161525 PMC4891303

[B39] Jing M, et al. (2020) An optimized acetylcholine sensor for monitoring in vivo cholinergic activity. Nat Methods 17:1139–1146. 10.1038/s41592-020-0953-2 32989318 PMC7606762

[B40] Khateb A, Mühlethaler M, Alonso A, Serafin M, Mainville L, Jones BE (1992) Cholinergic nucleus basalis neurons display the capacity for rhythmic bursting activity mediated by low-threshold calcium spikes. Neuroscience 51:489–494. 10.1016/0306-4522(92)90289-E 1488109

[B41] Kim R, Ananth MR, Desai NS, Role LW, Talmage DA (2024) Distinct subpopulations of ventral pallidal cholinergic projection neurons encode valence of olfactory stimuli. Cell Rep 43:114009. 10.1016/j.celrep.2024.114009 38536818 PMC11080946

[B42] Kim T, et al. (2015) Cortically projecting basal forebrain parvalbumin neurons regulate cortical gamma band oscillations. Proc Natl Acad Sci U S A 112:3535–3540. 10.1073/pnas.1413625112 25733878 PMC4371918

[B43] Király B, et al. (2023) The medial septum controls hippocampal supra-theta oscillations. Nat Commun 14:6159. 10.1038/s41467-023-41746-0 37816713 PMC10564782

[B44] Knowland D, Lilascharoen V, Pacia CP, Shin S, Wang EHJ, Lim BK (2017) Distinct ventral pallidal neural populations mediate separate symptoms of depression. Cell 170:284–297.e18. 10.1016/j.cell.2017.06.015 28689640 PMC5621481

[B45] Kupchik YM, Brown RM, Heinsbroek JA, Lobo MK, Schwartz DJ, Kalivas PW (2015) Coding the direct/indirect pathways by D1 and D2 receptors is not valid for accumbens projections. Nat Neurosci 18:1230–1232. 10.1038/nn.4068 26214370 PMC4551610

[B46] Kvitsiani D, Ranade S, Hangya B, Taniguchi H, Huang JZ, Kepecs A (2013) Distinct behavioural and network correlates of two interneuron types in prefrontal cortex. Nature 498:363–366. 10.1038/nature12176 23708967 PMC4349584

[B47] Larsen RS, Waters J (2018) Neuromodulatory correlates of pupil dilation. Front Neural Circuits 12:21. 10.3389/fncir.2018.00021 29593504 PMC5854659

[B48] Laszlovszky T, Schlingloff D, Hegedüs P, Freund TF, Gulyás A, Kepecs A, Hangya B (2020) Distinct synchronization, cortical coupling and behavioral function of two basal forebrain cholinergic neuron types. Nat Neurosci 23:992–1003. 10.1038/s41593-020-0648-0 32572235 PMC7611978

[B49] Lee CR, Margolis DJ (2016) Pupil dynamics reflect behavioral choice and learning in a Go/NoGo tactile decision-making task in mice. Front Behav Neurosci 10:200. 10.3389/fnbeh.2016.00200 27847470 PMC5088187

[B50] Lerner TN, et al. (2015) Intact-brain analyses reveal distinct information carried by SNc dopamine subcircuits. Cell 162:635–647. 10.1016/j.cell.2015.07.014 26232229 PMC4790813

[B51] Letzkus JJ, Wolff SBE, Meyer EMM, Tovote P, Courtin J, Herry C, Lüthi A (2011) A disinhibitory microcircuit for associative fear learning in the auditory cortex. Nature 480:331–335. 10.1038/nature10674 22158104

[B52] Lin S-C, Nicolelis MA (2008) Neuronal ensemble bursting in the basal forebrain encodes salience irrespective of valence. Neuron 59:138–149. 10.1016/j.neuron.2008.04.031 18614035 PMC2697387

[B53] Lovett-Barron M, Kaifosh P, Kheirbek MA, Danielson N, Zaremba JD, Reardon TR, Turi GF, Hen R, Zemelman BV, Losonczy A (2014) Dendritic inhibition in the hippocampus supports fear learning. Science 343:857–863. 10.1126/science.1247485 24558155 PMC4018419

[B54] Lozovaya N, Moumen A, Hammond C (2024) Basal forebrain cholinergic neurons have specific characteristics during the perinatal period. eNeuro 11:ENEURO.0538-23.2024. 10.1523/ENEURO.0538-23.2024 PMC11137802 38755010

[B55] Mathis A, Mamidanna P, Cury KM, Abe T, Murthy VN, Mathis MW, Bethge M (2018) DeepLabCut: markerless pose estimation of user-defined body parts with deep learning. Nat Neurosci 21:1281–1289. 10.1038/s41593-018-0209-y 30127430

[B56] Maurice N, Deniau JM, Menetrey A, Glowinski J, Thierry AM (1997) Position of the ventral pallidum in the rat prefrontal cortex-basal ganglia circuit. Neuroscience 80:523–534. 10.1016/S0306-4522(97)00002-X 9284354

[B57] McInnes L, Healy J, Melville J (2018) UMAP: uniform manifold approximation and projection for dimension reduction. arXiv:1–63.

[B58] Najafi F, Giovannucci A, Wang SSH, Medina JF (2014) Coding of stimulus strength via analog calcium signals in Purkinje cell dendrites of awake mice. Elife 3:e03663. 10.7554/eLife.03663 25205669 PMC4158287

[B59] Nelson A, Mooney R (2016) The basal forebrain and motor cortex provide convergent yet distinct movement-related inputs to the auditory cortex. Neuron 90:635–648. 10.1016/j.neuron.2016.03.031 27112494 PMC4866808

[B60] Neuhofer D, Kalivas P (2023) Differential modulation of GABAergic and glutamatergic neurons in the ventral pallidum by GABA and neuropeptides. eNeuro 10:ENEURO.0404-22.2023. 10.1523/ENEURO.0404-22.2023 PMC10348443 37414552

[B61] Neyhart E, et al. (2024) Cortical acetylcholine dynamics are predicted by cholinergic axon activity and behavior state. Cell Rep 43:114808. 10.1016/j.celrep.2024.114808 39383037 PMC11755675

[B62] Ottenheimer DJ, Bari BA, Sutlief E, Fraser KM, Kim TH, Richard JM, Cohen JY, Janak PH (2020a) A quantitative reward prediction error signal in the ventral pallidum. Nat Neurosci 23:1267–1276. 10.1038/s41593-020-0688-5 32778791 PMC7870109

[B63] Ottenheimer DJ, Wang K, Tong X, Fraser KM, Richard JM, Janak PH (2020b) Reward activity in ventral pallidum tracks satiety-sensitive preference and drives choice behavior. Sci Adv 6:27–30. 10.1126/sciadv.abc9321 PMC7673692 33148649

[B64] Ottenheimer D, Richard JM, Janak PH (2018) Ventral pallidum encodes relative reward value earlier and more robustly than nucleus accumbens. Nat Commun 9:4350. 10.1038/s41467-018-06849-z 30341305 PMC6195583

[B65] Pardo-Garcia TR, Garcia-Keller C, Penaloza T, Richie CT, Pickel J, Hope BT, Harvey BK, Kalivas PW, Heinsbroek JA (2019) Ventral pallidum is the primary target for accumbens D1 projections driving cocaine seeking. J Neurosci 39:2041–2051. 10.1523/JNEUROSCI.2822-18.2018 30622165 PMC6507080

[B66] Prasad AA, Xie C, Chaichim C, Nguyen JH, McClusky HE, Killcross S, Power JM, McNally GP (2020) Complementary roles for ventral pallidum cell types and their projections in relapse. J Neurosci 40:880–893. 10.1523/JNEUROSCI.0262-19.2019 31818977 PMC6975293

[B67] Ranjan R, Van Geit W, Moor R, Rössert C, Riquelme J, Damart T, Jaquier A, Tuncel A, Mandge D, Kilic I (2024) eFEL (5.7.13). Available at: 10.5281/zenodo.14222078

[B68] Reimer J, McGinley MJ, Liu Y, Rodenkirch C, Wang Q, McCormick DA, Tolias AS (2016) Pupil fluctuations track rapid changes in adrenergic and cholinergic activity in cortex. Nat Commun 7:13289. 10.1038/ncomms13289 27824036 PMC5105162

[B69] Richard JM, Ambroggi F, Janak PH, Fields HL (2016) Ventral pallidum neurons encode incentive value and promote cue-elicited instrumental actions. Neuron 90:1165–1173. 10.1016/j.neuron.2016.04.037 27238868 PMC4911300

[B70] Richard JM, Stout N, Acs D, Janak PH (2018) Ventral pallidal encoding of reward-seeking behavior depends on the underlying associative structure. Elife 7:1–25. 10.7554/eLife.33107 PMC5864276 29565248

[B71] Robert B, Kimchi EY, Watanabe Y, Chakoma T, Jing M, Li Y, Polley DB (2021) A functional topography within the cholinergic basal forebrain for encoding sensory cues and behavioral reinforcement outcomes. Elife 10:1–28. 10.7554/eLife.69514 PMC8654357 34821218

[B72] Root DH, Melendez RI, Zaborszky L, Napier TC (2015) The ventral pallidum: subregion-specific functional anatomy and roles in motivated behaviors. Prog Neurobiol 130:29–70. 10.1016/j.pneurobio.2015.03.005 25857550 PMC4687907

[B73] Royer S, Zemelman BV, Losonczy A, Kim J, Chance F, Magee JC, Buzsáki G (2012) Control of timing, rate and bursts of hippocampal place cells by dendritic and somatic inhibition. Nat Neurosci 15:769–775. 10.1038/nn.3077 22446878 PMC4919905

[B74] Saga Y, Richard A, Sgambato-Faure V, Hoshi E, Tobler PN, Tremblay L (2017) Ventral pallidum encodes contextual information and controls aversive behaviors. Cereb Cortex 27:2528–2543. 10.1093/cercor/bhw107 27114173

[B75] Saper CB (1984) Organization of cerebral cortical afferent systems in the rat. II. Magnocellular basal nucleus. J Comp Neurol 222:313–342. 10.1002/cne.902220302 6699210

[B76] Schmitzer-Torbert N, Jackson J, Henze D, Harris K, Redish AD (2005) Quantitative measures of cluster quality for use in extracellular recordings. Neuroscience 131:1–11. 10.1016/j.neuroscience.2004.09.066 15680687

[B77] Smith KS, Tindell AJ, Aldridge JW, Berridge KC (2009) Ventral pallidum roles in reward and motivation. Behav Brain Res 196:155–167. 10.1016/j.bbr.2008.09.038 18955088 PMC2606924

[B78] Soares-Cunha C, Domingues AV, Correia R, Coimbra B, Vieitas-Gaspar N, de Vasconcelos NAP, Pinto L, Sousa N, Rodrigues AJ (2022) Distinct role of nucleus accumbens D2-MSN projections to ventral pallidum in different phases of motivated behavior. Cell Rep 38:110380. 10.1016/j.celrep.2022.110380 35172164 PMC8864463

[B79] Soares-Cunha C, Heinsbroek JA (2023) Ventral pallidal regulation of motivated behaviors and reinforcement. Front Neural Circuits 17:1–25. 10.3389/fncir.2023.1086053 PMC9932340 36817646

[B80] Solari N, Sviatkó K, Laszlovszky T, Hegedüs P, Hangya B (2018) Open source tools for temporally controlled rodent behavior suitable for electrophysiology and optogenetic manipulations. Front Syst Neurosci 12:18. 10.3389/fnsys.2018.00018 29867383 PMC5962774

[B81] Stephenson-Jones M, Bravo-Rivera C, Ahrens S, Furlan A, Xiao X, Fernandes-Henriques C, Li B (2020) Opposing contributions of GABAergic and glutamatergic ventral pallidal neurons to motivational behaviors. Neuron 105:921–933.e5. 10.1016/j.neuron.2019.12.006 31948733 PMC8573387

[B82] Sturgill JF, Hegedus P, Li SJ, Chevy Q, Siebels A, Jing M, Li Y, Hangya B, Kepecs A (2020) Basal forebrain-derived acetylcholine encodes valence-free reinforcement prediction error. bioRxiv.

[B83] Szymusiak R, Alam N, McGinty D (2000) Discharge patterns of neurons in cholinergic regions of the basal forebrain during waking and sleep. Behav Brain Res 115:171–182. 10.1016/S0166-4328(00)00257-6 11000419

[B84] Tachibana Y, Hikosaka O (2012) The primate ventral pallidum encodes expected reward value and regulates motor action. Neuron 76:826–837. 10.1016/j.neuron.2012.09.030 23177966 PMC3519929

[B85] Tindell AJ (2004) Ventral pallidal representation of Pavlovian cues and reward: population and rate codes. J Neurosci 24:1058–1069. 10.1523/JNEUROSCI.1437-03.2004 14762124 PMC6793590

[B86] Tindell AJ, Berridge KC, Zhang J, Peciña S, Aldridge JW (2005) Ventral pallidal neurons code incentive motivation: amplification by mesolimbic sensitization and amphetamine. Eur J Neurosci 22:2617–2634. 10.1111/j.1460-9568.2005.04411.x 16307604

[B87] Tindell AJ, Smith KS, Peciña S, Berridge KC, Aldridge JW (2006) Ventral pallidum firing codes hedonic reward: when a bad taste turns good. J Neurophysiol 96:2399–2409. 10.1152/jn.00576.2006 16885520

[B88] Tripathi A, Prensa L, Mengual E (2013) Axonal branching patterns of ventral pallidal neurons in the rat. Brain Struct Funct 218:1133–1157. 10.1007/s00429-012-0451-0 22932869

[B89] Unal CT, Golowasch JP, Zaborszky L (2012) Adult mouse basal forebrain harbors two distinct cholinergic populations defined by their electrophysiology. Front Behav Neurosci 6:21. 10.3389/fnbeh.2012.00021 22586380 PMC3346982

[B90] van den Bos R, Cools AR (1991) Motor activity and the GABAA-receptor in the ventral pallidum/substantia innominata complex. Neurosci Lett 124:246–250. 10.1016/0304-3940(91)90105-3 1648694

[B91] Walaas I, Fonnum F (1979) The distribution and origin of glutamate decarboxylase and choline acetyltransferase in ventral pallidum and other basal forebrain regions. Brain Res 177:325–336. 10.1016/0006-8993(79)90783-2 497834

[B92] Wassum KM, Ostlund SB, Maidment NT, Balleine BW (2009) Distinct opioid circuits determine the palatability and the desirability of rewarding events. Proc Natl Acad Sci U S A 106:12512–12517. 10.1073/pnas.0905874106 19597155 PMC2718390

[B93] Wulff AB, Tooley J, Marconi LJ, Creed MC (2019) Ventral pallidal modulation of aversion processing. Brain Res 1713:62–69. 10.1016/j.brainres.2018.10.010 30300634

[B94] Yang C, Thankachan S, McCarley RW, Brown RE (2017) The menagerie of the basal forebrain: how many (neural) species are there, what do they look like, how do they behave and who talks to whom? Curr Opin Neurobiol 44:159–166. 10.1016/j.conb.2017.05.004 28538168 PMC5525536

[B95] Zaborszky L, Gaykema R, Swanson D, Cullinan W (1997) Cortical input to the basal forebrain. Neuroscience 79:1051–1078. 10.1016/S0306-4522(97)00049-3 9219967

[B96] Zaborszky L, van den Pol A, Gyengesi E (2012) The basal forebrain cholinergic projection system in mice. In: The mouse nervous system (Watson C, Paxinos G, Puelles L, eds), Ed 1, pp 684–718. Amsterdam: Elsevier.

[B97] Záborszky L, Cullinan WE (1992) Projections from the nucleus accumbens to cholinergic neurons of the ventral pallidum: a correlated light and electron microscopic double-immunolabeling study in rat. Brain Res 570:92–101. 10.1016/0006-8993(92)90568-T 1617433

[B98] Zahm DS, Williams E, Wohltmann C (1996) Ventral striatopallidothalamic projection: IV. Relative involvements of neurochemically distinct subterritories in the ventral pallidum and adjacent parts of the rostroventral forebrain. J Comp Neurol 364:340–362. 10.1002/(SICI)1096-9861(19960108)364:2<340::AID-CNE11>3.0.CO;2-T 8788254

[B99] Zhang Y, Reynolds JNJ, Cragg SJ (2018) Pauses in cholinergic interneuron activity are driven by excitatory input and delayed rectification, with dopamine modulation. Neuron 98:918–925.e3. 10.1016/j.neuron.2018.04.027 29754751 PMC5993868

